# Unproductive alternative splicing of *ATM* exon 7: mapping of critical regulatory elements and identification of 34 spliceogenic variants

**DOI:** 10.1007/s00109-025-02595-0

**Published:** 2025-09-20

**Authors:** Inés Llinares-Burguet, Lara Sanoguera-Miralles, Alicia García-Álvarez, Ada Esteban-Sánchez, María José Caloca, Miguel de la Hoya, Elena Bueno-Martínez, Eladio A. Velasco-Sampedro

**Affiliations:** 1 Splicing and Genetic Susceptibility to Cancer, Unidad de Excelencia Instituto de Biomedicina y Genética Molecular de Valladolid (IBGM), Universidad de Valladolid – Consejo Superior de Investigaciones Científicas (UVa-CSIC), Sanz y Forés 3, 47003 Valladolid, Spain; 2 Molecular Oncology Laboratory, Hospital Clínico San Carlos, IdISSC (Instituto de Investigación Sanitaria del Hospital Clínico San Carlos), Madrid, Spain; 3 Rho GTPasas y Señalización Por Lípidos, Unidad de Excelencia Instituto de Biomedicina y Genética Molecular de Valladolid (IBGM), Universidad de Valladolid – Consejo Superior de Investigaciones Científicas (UVa-CSIC), Valladolid, Spain

**Keywords:** Hereditary breast cancer, *ATM*, Variants of uncertain significance, Unproductive splicing, Aberrant splicing, Splicing regulatory elements, Minigenes

## Abstract

**Abstract:**

*ATM* loss-of-function variants are significantly associated with increased breast cancer risk. *ATM* exon 7 skipping (△(E7)) is a naturally-occurring alternative splicing event, which introduces a premature termination codon and represents a form of gene-expression regulation via unproductive splicing. Disruption of splicing regulatory elements (SRE) by variants can lead to mis-splicing, potentially contributing to disease susceptibility. To study the regulatory mechanisms of △(E7) and the impact of exonic variants on splicing, a combined in silico/minigene approach was employed, using the construct mgATM_4-9 (exons 4–9) that recapitulates this splicing event. HEXplorer analysis of *ATM* exon 7 predicted two splicing enhancer-rich regions (c.665–681 and c.867–898). Deletions of these intervals in mgATM_4-9 significantly increased △(E7) (57–96%), revealing their critical role for exon 7 inclusion. Forty-eight candidate variants (HEXplorer, △HZEI score < -40) within these SRE-rich segments were functionally assayed, 34 of which (71%) impaired exon 7 recognition. Nineteen variants presented strong impacts with high expression of △(E7)-transcripts (69–96%), of which c.668A > T, c.680C > A and c.680C > T exhibited particularly strong effects (4–13% full-length transcripts). DeepCLIP analysis suggested that SR proteins SRSF7 and SRSF10 play a positive regulatory role in exon 7 inclusion. Eight variants were classified as likely pathogenic according to ACMG/AMP-based guidelines. Furthermore, nine missense and two synonymous variants with strong impacts (16–29% full-length transcripts) might represent intermediate risk alleles. This work collectively demonstrates the intricate nature of *ATM* exon 7 recognition, regulated by *cis*-acting SREs, emphasizing the value of in silico predictions for initial variant filtering and minigene assays for dissecting splicing regulation and clinical interpretation of variants.

**Key Messages:**

- Frameshift *ATM* exon 7 skipping is an unproductive alternative splicing event that may represent a form of gene-expression regulation.
- Two SRE-rich intervals are involved in exon 7 recognition.
- Thirty-four out of 48 tested variants located at these intervals disrupted exon 7 inclusion. Nineteen variants showed strong impacts on splicing.
- All variant types can impair splicing: 23 missense, 7 nonsense, and 4 synonymous variants upregulate exon 7 skipping.
- Eight variants were classified as likely pathogenic.

**Supplementary Information:**

The online version contains supplementary material available at 10.1007/s00109-025-02595-0.

## Introduction

The ataxia telangiectasia-mutated (*ATM*) gene (MIM# 607,585) encodes a large serine/threonine protein kinase that belongs to the phosphatidylinositol 3′-kinase (PI3K)-related kinase (PIKK) family. This protein plays an important role in the double-strand DNA break response, phosphorylating effectors in signal transduction pathways that ultimately induce cell cycle arrest, DNA repair or cell death and, therefore, it is crucial for genomic integrity [1–3].

Biallelic loss-of-function variants in this gene result in ataxia-telangiectasia syndrome, a rare neurodegenerative disease [4]. Additionally, carriers of monoallelic variants face elevated risks for several adult-onset cancers, including breast, pancreatic, gastric, and prostate cancers [5]. More precisely, carriers have a 6.5-fold increase for pancreatic cancer [6], and female carriers have approximately a twofold increased lifetime risk of estrogen receptor–positive breast cancer, with a penetrance of 20–30% [7–10]. The overall frequency of pathogenic variants in breast cancer patients is approximately 0.7% [8, 9], with significant differences observed between non-Hispanic White and Asian populations [11]. The National Comprehensive Cancer Network guidelines (v1.2026; https://www.nccn.org/guidelines/guidelines-detail?category=2&id=1545, accessed August 21, 2025) recommend annual mammography at age 40 and breast magnetic resonance imaging at age 30 to 35 in carriers of *ATM* pathogenic variants. This strategy may reduce breast cancer mortality by ~ 60% for women with *ATM* pathogenic variants [12].

Splicing is a crucial step in the gene expression process of eukaryotic cells, wherein intron sequences are precisely removed from precursor mRNA, and consecutive exons are joined to produce mature mRNA. This operation is carried out by the spliceosome, a complex machinery that specifically recognizes key conserved sequences, such as splice-sites (5′ss or donor and 3’ss or acceptor), the polypyrimidine tract and the branch point. Furthermore, a myriad of exonic and intronic *cis*-regulatory sequences (splicing regulatory elements, SREs) promote (enhancers, ESE/ISE) or repress (silencers, ESS/ISS) exon inclusion by recruiting *trans*-acting proteins, called splicing factors. All these elements play a major role in splicing regulation, including the recognition of alternative exons, non-canonical splice-sites, and exons of atypical size, among other functions [13]. Therefore, there are a wide array of splicing motifs that are major targets for potential spliceogenic variants that might cause a genetic disorder [14–16]. In fact, splicing dysfunction by germline variants is a relevant mechanism of pathogenicity in disease genes [17–19]. Direct analysis of patient RNA is recommended to evaluate the splicing impact of variants [20]. However, this is often not practical in a diagnostic setting because blood RNA samples may not always be available. Alternatively, splicing reporter minigenes are a simple and robust method for assessing candidate variants [21, 22].

Over the years, several in silico models have been developed to predict genetic variants leading to aberrant splicing. Since donor and acceptor sites are reasonably conserved, alterations can be fairly predicted by these tools. However, those targeting the much less conserved SREs are less successful, and their results should be interpreted with caution. As an alternative to the in silico strategy, we have previously demonstrated that minigene-based technologies are very useful for mapping SRE-rich intervals using internal exonic microdeletions [23–25].

*ATM* exon 7 skipping (△(E7)) is a frequent alternative splicing (AS) event detected in 13% of SpliceVault samples (https://kidsneuro.shinyapps.io/splicevault/; accessed on 20/08/2025)[26]. △(E7) introduces a premature termination codon (PTC), predicted to elicit the Nonsense-Mediated Decay (NMD) pathway. Thus, exon 7 skipping may represent the so-called unproductive splicing (or AS-NMD) that constitutes a form of gene-expression regulation [27]. This study primarily focuses on mapping the *cis*-acting regulatory elements involved in exon 7 recognition. To this end, we developed a combined approach of bioinformatics tools and minigene assays. Then, 48 exonic variants located within ESE-rich intervals were selected for RNA analysis. For these purposes, we employed a previously published minigene encompassing *ATM* exons 4–9, where 14 splice-site variants had been previously characterized [28].

## Materials and methods

Ethical approval for this study was obtained from the Ethics Committee of the Spanish National Research Council-CSIC (28/05/2018).

### Annotation

Variants, microdeletions, transcripts and predicted protein products were annotated following the Human Genome Variation Society (HGVS) guidelines, based on the *ATM* MANE select transcript (NM_000051.4) composed of 63 exons. To simplify, splicing isoforms were described using abbreviated notations as follows: ▼ (incorporation of intronic sequences), △ (deletion of exonic sequences), E (exon), p (alternative acceptor site), q (alternative donor site) and a number representing the exact number of nucleotides incorporated or skipped [29].

### Bioinformatics and variant selection

Exon 7 sequence and variants were bioinformatically analyzed using two in silico tools:

1. HEXplorer (https://rna.hhu.de/HEXplorer/) was used to predict enhancer and silencer-rich regions in a particular genomic sequence and to select candidate spliceogenic variants by calculating the difference of the HEXplorer score (△HZEI) between the mutant and reference sequences [30]. While other studies had used HEXplorer score cut-offs of ≤ −5 or ≤ −20 [31–33], our previous analysis of 87 *CHEK2* variants revealed that a more stringent cut-off of ≤ −40 would have been more effective in filtering out a significant number of false positive and low-splicing impact variants. We therefore established △HZEI ≤ −40 as the cut-off for our analysis [25].
2. SpliceAI (https://spliceailookup.broadinstitute.org/) is a deep learning-based approach used to predict the impact of genetic variants on RNA splicing [34]. In this study, SpliceAI was employed to predict splicing outcomes for 147 *ATM* variants.

We also used the deep learning approach DeepCLIP (https://deepclip-web.compbio.sdu.dk/) [35] to predict the effect of spliceogenic variants on protein-RNA binding. Average scores of the different DeepCLIP models were calculated for wild type (wt) and mutant sequences. Then, a △ score was computed, and a threshold of ± 0.2 was assumed as an indicative score to identify putative splicing factors involved in exon recognition.

### Minigene construction and site-directed mutagenesis

Minigene assays have been performed using a previously published minigene (mgATM_4-9) including an insert with *ATM* exons 4–9 and approximately 200 nucleotides of flanking intronic sequences upstream and downstream from each exon, subcloned into the splicing plasmid pSAD (Patent P201231427-CSIC) [22] (Supplementary Figure S1).

Two microdeletions, 48 exonic and two splice-site single-nucleotide variants were separately introduced into the wt minigene by site-directed mutagenesis using the QuikChange Lightning kit (Agilent, Santa Clara, CA) (Supplementary Table S1). All constructs were confirmed by Sanger sequencing (Macrogen).

### Functional assays

For the minigene assays, we principally used the MCF-7 cell line (ATCC HTB-22; LGC Standards, Barcelona, Spain) [22]. MCF-7 is an estrogen receptor-positive breast cancer cell line, like most *ATM*-associated breast tumors. Around 2 × 10^5^ MCF-7 cells were grown to 90% confluence in 0.5 ml of Gibco™ MEM medium supplemented with 10% fetal bovine serum, 1% non-essential amino acids and 1% penicillin/streptomycin stock solution (ThermoFisher Scientific, Waltham, MA) using 4-well plates (Nunc, Roskilde, Denmark). SK-BR-3 (ATCC HBT-30; LGC Standards) and HMEC (primary Human Mammary Epithelial Cells; ThermoFisher Scientific) were also used to check reproducibility of splicing assays. HMEC cells were specifically grown in 0.5 mL of HuMEC Basal Medium (ThermoFisher Scientific) supplemented with 5 mL of HuMEC Supplement, 2% fetal bovine serum and 1% penicillin/streptomycin.

**Fig. 1:**
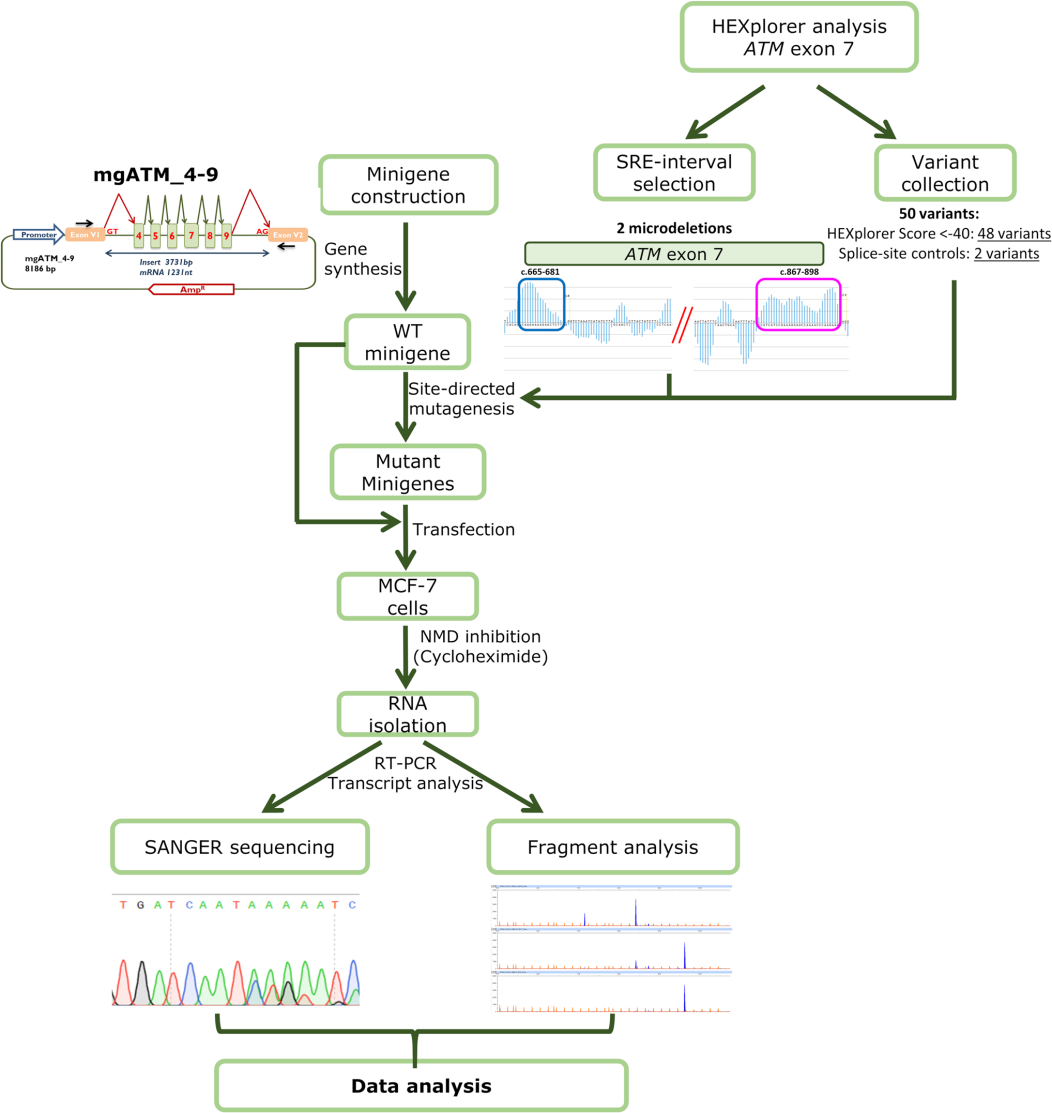
Workflow of the minigene protocol for *ATM* exon 7. The basic assay includes the following steps: 1) Minigene construction; 2) Bioinformatics analysis of splicing regulatory motifs; 3) Site-directed mutagenesis; 4) Transfection of the wild type and mutant minigenes; 5) Inhibition of Nonsense-mediated decay and RNA purification; 6) Transcript sequencing and fragment analysis by fluorescent capillary electrophoresis; 7) Data interpretation

Two microlitres of Lipofectamine LTX (Life Technologies, Carlsbad, CA) were used to transiently transfect cells with 1 μg of each minigene. NMD was inhibited by incubation with cycloheximide 300 μg/ml (Sigma-Aldrich, St. Louis, MO) for 4 h before RNA extraction. RNA was purified using the Genematrix Universal RNA Purification Kit (EURx, Gdansk, Poland) following the manufacturer's instructions with on-column DNAse I digestion. Then, 400 ng of each sample RNA were retrotranscribed using the RevertAid First-Strand cDNA Synthesis Kit (Life Technologies, Carlsbad, CA, USA) and the minigene exon V2-specific primer RTPSPL3-RV (5′-TGAGGAGTGAATTGGTCGAA-3′). Subsequently, 40 ng of cDNA were used for amplifying the regions of interest using the vector-specific primers RTPSPL3-FW (5'- TCACCTGGACAACCTCAAAG-3') and RTpSAD-RV (Patent P201231427) and Platinum Taq polymerase (Life Technologies). Samples were denatured at 94ºC/2 min, followed by 35 cycles of 94ºC/30 s, 60ºC/30 s, and 72ºC (1 min/kb), and a final extension step at 72ºC/5 min. RT-PCR products were sequenced at the Macrogen facility. The expected minigene full-length (mgFL) transcript is 1231 nt long. In order to estimate the relative contribution of each transcript semi-quantitative fluorescent RT-PCRs were performed in triplicate using RTPSPL3-FW and FAM-labelled RTpSAD-RV primers under standard conditions, except that the number of cycles was reduced to 26. Fluorescent products were run with LIZ-1200 Size Standard at the Macrogen facility (Seoul, Korea) and analyzed using Peak Scanner software V1.0 (Life Technologies) [22, 36]. Only peak heights ≥ 50 RFU (Relative Fluorescence Units) were considered. The mean peak areas from three separated experiments for each variant were utilized to calculate both the relative ratios of each transcript and their corresponding standard deviations. The procedure is outlined in Fig. 1.

## Results

**Fig. 2:**
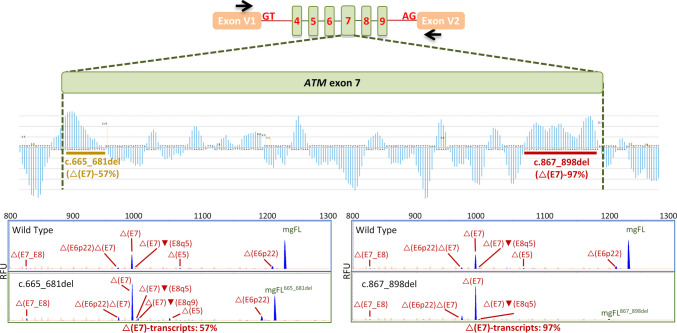
SRE profile, map and splicing functional assays of microdeletions of *ATM* exon 7. Top: schematic representation of mgATM_4-9 insert. Middle: HEXplorer profile and map of microdeletions introduced into the wild type minigene (red line, strong impact on splicing; orange, moderate impact). Bottom: fluorescent fragment analysis of transcripts generated by wild type and microdeletion minigenes. FAM-labeled products (blue peaks) were run with LIZ-1200 (orange peaks) as size standard. RFU (y-axis), relative fluorescent units; mgFL, minigene full-length

As previously published [28], the wt minigene mgATM_4-9 produced the expected mgFL transcript (1231-nt) and the alternative isoform △(E7) as main outcomes (57.6% and 30.6% respectively). The minigene therefore mimicked the splicing patterns observed in healthy breast tissue (Supplementary Figure S2**A**), confirming it as a suitable tool for assessing *ATM* exon 7 splicing. The role of this alternative event, if any, is unknown as the loss of exon 7 (239 bp; r.663_901del) is predicted to truncate ATM (p.Gln222Cysfs*3) and trigger NMD. In addition, other transcripts were expressed at low levels: △(E6p22) (5.5%), [△(E7)▼(E8q5)] (1%), △(E5) (1.3%), [△(E6p22)△(E7)] (2.8%) and △(E7_E8) (1.2%) (Fig. 2**; **Table 1**; **Supplementary Table S2). All the alternative events, including exon 7 skipping, are abolished in the presence of the DNA damaging agent cisplatin (Supplementary Figure S2**B-C**), suggesting that DNA damage response (DDR) up-regulates *ATM* expression by reducing AS-NMD. In fact, cisplatin was previously reported to modify 717 splicing events (including 245 cassette exons) via the splicing factor SRSF4 [37].

**Table 1:** Splicing outcomes of microdeletions and SRE-variants of *ATM* exon 7

Microdel	HEXplorer Score	mgFL-transcripts	Exon 7-transcripts^1^	Other transcripts^2^
**mgATM_4-9**		57.6%	35.6%	6.8%
c.665_681del		34.6%	56.7%	8.7%
c.867_898del		2.8%	97.2%	-
**Variants**^**3**^				
*c.663-2A > G		-	100%	-
c.665A > T	−117.5	48.8%	47.2%	4%
c.666A > C^4^	−55.6	81.5%	11.5%	7%
c.666A > G	−65.6	63%	34.8%	2.2%
c.666A > T^4^	−64.8	84.8%	8%	7.2%
*c.667G > A	−113	32.4%	66.3%	1.3%
*c.667G > C	−40.6	23.1%	74.4%	2.5%
*c.667G > T	−136.6	25.4%	71.1%	3.5%
c.668A > G	−42.1	66.2%	28%	5.8%
*c.668A > T	−112.1	12.9%	87.1%	-
*c.669A > T	−84.4	17.6%	81%	1.4%
*c.670A > T	−99.5	24.1%	72.5%	3.4%
*c.671A > T	−42.4	45.8%	50.9%	3.3%
*c.672G > T	−103.4	25.8%	72.2%	2%
*c.673A > G	−72.8	37%	60.3%	2.7%
c.673A > T	−45.5	65.5%	29.3%	5.2%
*c.677C > T	−93.4	19.1%	79.6%	1.3%
*c.680C > A	−53.3	5.5%	91%	3.5%
*c.680C > T	−90.9	3.9%	96.1%	-
*c.868C > T	−48.2	43.1%	56.9%	-
*c.869A > C	−55.8	33.8%	63.7%	2.5%
*c.869A > T	−42.2	44.5%	52.5%	3%
*c.871C > A	−95.8	25.8%	72.7%	1.5%
*c.871C > T	−72.6	29.1%	68.9%	2%
*c.872A > C	−45.1	31.4%	68.6%	-
*c.872A > T	−44.7	43%	52.4%	4.6%
*c.874C > A	−50.4	36.9%	59.3%	3.8%
c.875C > T	−43.1	56.4%	38.7%	4.9%
*c.876G > T	−62.9	47.2%	50%	2.8%
*c.877A > T	−45.6	27.8%	69%	3.2%
*c.878A > T	−66.8	29.2%	69%	1.8%
c.879A > G	−69.2	60.4%	33.2%	6.4%
*c.881G > T	−79	15.9%	84.1%	**-**
*c.882A > G	−99.8	24.5%	73.9%	1.6%
*c.882A > T	−46.6	26.7%	71.5%	1.8%
*c.885C > T	−40.6	36.2%	61%	2.8%
*c.886A > T	−54.4	30.7%	64.8%	4.5%
*c.887A > T	−46.1	45.1%	50.4%	4.5%
*c.892C > T	−71.5	21.5%	77.2%	1.3%
c.893A > C	−43	52.9%	35.1%	12%
*c.893A > T	−104.7	27.1%	70.4%	2.5%
c.894A > C^4^	−43.3	75.7%	16%	8.3%
c.894A > G^4^	−46.8	71.5%	21%	7.5%
c.894A > T	−59.2	50.7%	42.1%	7.2%
c.895G > A	−126.2	58.3%	40.1%	1.6%
*c.895G > T	−138	25.4%	63.4%	11.2%
*c.896A > T	−116	44.9%	50.9%	4.2%
c.897A > T	−67.5	50.5%	44.2%	5.3%
*c.898A > T	−77.5	37.7%	56.1%	6.2%
*c.901 + 2 T > C^**5**^		-	100%	-

^1^ Exon 7-transcripts: Transcripts with splicing events affecting exon 7 were grouped. This group of transcripts includes: △(E7), △(E6p22)△(E7), △(E7)▼(E8q5), △(E7_E8), △(E7)▼(E8q9), ▼(E7p79) and ▼(E7q283^mg^). The specific contribution and standard deviations of each transcript are detailed in Supplementary Table S2

^2^ Other transcripts: △(E6p22) (the most abundant), △(E5) and the uncharacterized isoform of 861-nt

^3^ Spliceogenic variants (mgFL < 47.6%) are shown with an asterisk. Variants with < 30% mgFL-transcript expression level are shown in bold

^4^ Variants with relevant increments of mgFL-transcripts (> 70% of the overall expression)

^5^ Bueno-Martínez et al. (2022) [28]

### SRE mapping

HEXplorer analysis identified two putative ESE-regions within the *ATM* exon 7: a 17-nt segment located at the 5’ end (c.665–681) and a 32-nt segment (c.867–898) at the 3’ end (Fig. 2). Two microdeletions covering these intervals, c.665_681del and c.867_898del, were introduced in the minigene by site-directed mutagenesis and a functional study was performed to confirm in silico data. Both microdeletions showed strong impact and a significant reduction of exon 7 inclusion, confirming the HEXplorer profile and the high density of ESEs. Deletion c.867_898del presented the strongest impact with only 2.8% of mgFL-transcripts suggesting that it is particularly enriched in SREs (Fig. 2**; **Table 1). Interestingly, unlike in the wt minigene, cisplatin did not decrease △(E7) transcript in c.867_898del microdeletion minigenes tested in MCF-7 cells, confirming that this interval contains target regulatory binding motifs of key splicing factors (Supplementary Figure S2**B**). Cisplatin has a moderate effect on exon 7 recognition in the c.665_681del-minigene (△(E7)-transcripts from 58.5% to 39.3%).

### Splicing assays of ESE variants

**Fig. 3:**
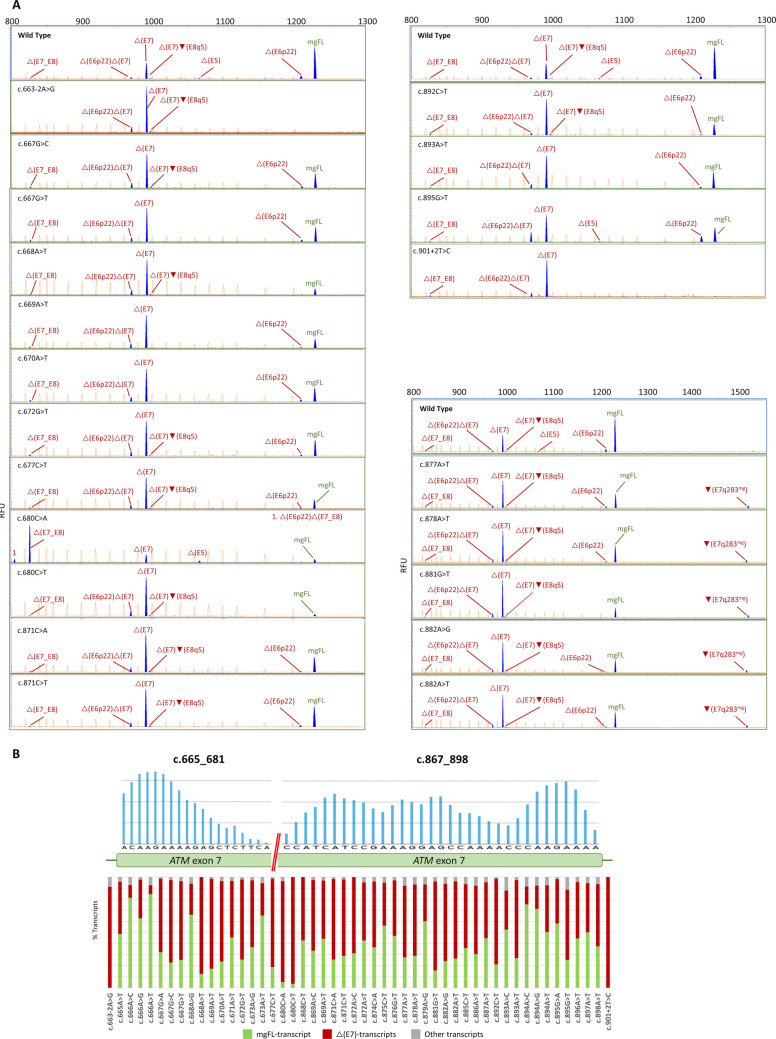
Splicing outcomes of spliceogenic variants. **A**) Electropherograms of spliceogenic variants with strong impacts on splicing; FAM-labeled products (blue peaks) were run with LIZ-1200 (orange peaks) as size standard. RFU (y-axis), relative fluorescent units; mgFL, minigene full-length. To visualize the Δ(E7q283^mg^) transcript, electropherograms of variants c.877A > T, c.878A > T, c.881G > T, c.882A > G and c.882A > T (right) are presented with the x-axis ranging from 800 to 1500 bp. B) Graphical representation of splicing outcomes. Bar graphs indicate the relative proportions of transcripts. All transcripts involving anomalous splicing events of exon 7 are grouped as ∆(E7)-transcripts (red bar). Other transcripts: transcripts that contain exon 7 (non-skipping of target exon)

We analyzed two splice-site variants as controls, c.663-2A > G (3’ss) and c.901 + 2 T > C (5’ss). Both variants mainly showed exon 7 skipping and no traces of mgFL-transcript (Fig. 3**; **Table 1**; **Supplementary Table S2).

All the possible single nucleotide substitutions of the presumed c.665–681 and c.867–898 ESE-rich intervals were analyzed by HEXplorer to detect putative spliceogenic variants. Out of the 147 analyzed changes, 48 showed △HZEI lower than −40 (Supplementary Table S3**; **Table 1), which were introduced into mgATM_4-9 for studying in MCF-7 cells. Operationally, variants were considered spliceogenic when they showed at least a 10% reduction in the overall expression of mgFL-transcript (cut-off ≤ 47.6%; note that spliceogenicity is not necessarily equivalent to pathogenicity). A total of 34 variants (70.8%) impaired splicing, nineteen (6 nonsense, 11 missense and 2 synonymous) of which exhibited higher levels of exon 7 skipping (up to 96.1%) and less than 30% mgFL-transcript. Remarkably, variants c.668A > T, c.680C > A and c.680C > T strongly impaired exon 7 recognition, drastically reducing mgFL-transcript expression to 12.9%, 5.5% and 3.9%, respectively, below our previously established loss-of-function threshold of 13% (Table 1**; **Fig. 3**; **Supplementary Figure S3) [28]. Finally, four variants at two different cDNA positions (c.666 and c.894) notably increased splicing efficiency (mgFL: 72–85%) and exon 7 inclusion (c.666A > C, c.666A > T, c.894A > C and c.894A > G; Table 1). Analysis of variants c.680C > T, c.881C > T and c.894A > C in SK-BR-3 and HMEC cells showed similar splicing outcomes (Supplementary Figure S3C).

Transcript analysis revealed that most variants typically yielded the same transcripts of the wt minigene, but at different expression levels. Interestingly, a new outcome with a minor contribution (≤ 4.6%), ▼(E7q283^mg^) (retention of 283-nt of the shortened minigene intron 7), was detected in variants c.877A > T, c.878A > T, c.881G > T, c.882A > G, c.882A > T, c.885C > T and c.886A > T, suggesting that these variants have a direct influence on 5’ss recognition (Fig. 3B**; **Table 1). It is also worth mentioning that c.680C > A is the only variant that resulted in skipping of exons 7 and 8 (△(E7_E8); 70% of the overall expression) as the predominant transcript. Conversely, c.680C > T (at the same position) mostly generated exon 7 skipping alone (80%; Supplementary Table S2). Finally, one minor uncharacterized isoform (861-nt) was detected, accounting for 2.4% of the overall expression.

In order to identify the putative splicing factors involved in exon 7 recognition, a DeepCLIP analysis was performed (Supplementary Table S4). In general, the analysis showed a decreased binding score in activator proteins, whereas hnRNPs proteins, typically splicing repressors, increased their binding affinity for most variants with less than 30% of mgFL-transcript. Particularly, DeepCLIP output suggests the loss of binding sites for SRSF7 (10 variants) and/or SRSF10 (8 variants) among the variants with the strongest effects. SRSF7, a splicing factor involved in alternative splicing and identification of non-canonical GC-5’ss [38, 39], acts as a splicing activator when recruited upstream of the 5’ss, similar to other SR proteins [40]. SRSF10 has a dual role as splicing repressor/activator function depending on its phosphorylation state and, interestingly, is involved in the regulation of the DDR [41–43]. However, these in silico predictions should be supported by protein-RNA binding assays. On the other hand, no significant changes were observed in binding capacities for variants c.666A > C,T and c.894A > C,G that increased splicing efficiency of exon 7 (data not shown).

## Discussion

Alternative splicing plays a pivotal role in increasing proteome diversity and control of gene expression in eukaryotes [13]. It is a highly regulated mechanism so that several elements are implicated in exon recognition through a combinatorial process, including: splice-site strength, a vast array of *cis*-acting sequences, *trans*-splicing factors, the genomic environment, pre-mRNA secondary structure, RNA polymerase II kinetics and epigenetic mechanisms [44, 45]. Consequently, genetic variants can frequently modify any of these factors making them significant contributors to Human genetic diseases, with estimates ranging from 15 to 60% of the pathogenic variants depending on the disease gene [17, 18, 46, 47]. Hence, splicing disruptions could represent a relevant deleterious mechanism for the vast amount of variants of uncertain significance (VUS) in the *ATM* gene (8169 VUS; https://www.ncbi.nlm.nih.gov/clinvar/, accessed 27/08/2025) [19, 48].

Here, we have performed an extensive analysis of the alternatively spliced *ATM* exon 7 by minigene assays of the mgATM_4-9 construct, through which two critical splicing regulatory regions and 34 spliceogenic variants were discovered. Remarkably, all variant types can impair splicing: 23 missense, 7 nonsense, and 4 synonymous variants upregulated exon 7 skipping.

**Fig. 4:**
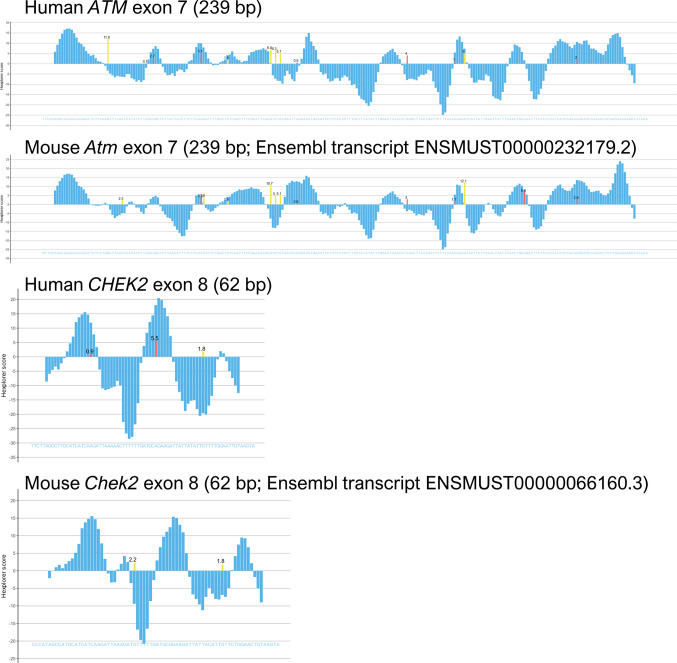
HEXplorer profiles of Human and mouse *ATM/Atm* exon 7 and *CHEK2/Chek2* exon 8. *ATM* and *CHEK2* exons plus seven 5’ and 3’ flanking intronic nucleotides were analyzed with HEXplorer (https://rna.hhu.de/HEXplorer/)

Exon 7 skipping in the *ATM* gene is a common naturally-occurring AS event [26]. This skipping introduces a PTC (p.(Gln222Cysfs*3)) and activates NMD, resembling unproductive splicing or AS-NMD, a cellular mechanism that down-regulates gene expression and protein production by targeting these transcripts for degradation [27]. Unproductive splicing affects about 15% of transcripts from protein coding genes [27], suggesting that AS-NMD is a widespread mechanism for gene expression regulation. Interestingly, some splicing factors utilize this process to autoregulate their expression levels through the introduction of “poison cassette exons” [49]. Given that *ATM* exon 7 skipping is completely abolished in DNA damage conditions of MCF-7 cells (Supplementary Figure S2**B**), we postulate that the DDR up-regulates fully functional ATM expression by impairing an AS-NMD event. Theoretically, a fully-functional ATM is essential for the DNA repair pathway due to its pivotal role in orchestrating the cellular response to DNA double-strand breaks [50]. Similarly, AS-NMD of exon 8 (p.(Pro283Aspfs*8)) in *CHEK2*, a downstream target of ATM in the DDR, may also be considered unproductive splicing to control CHK2 expression levels. Moreover, we have shown that both AS-NMD events are regulated by specific exonic sequences. Furthermore, the human and mouse HEXplorer profiles of *ATM* exon 7 and *CHEK2* exon 8 (Fig. 4), respectively, are highly similar suggesting that this regulatory mechanism is conserved across mammals. Furthermore, DeepCLIP analysis revealed that most variants disrupted binding sites for the SR proteins SRSF7 and SRSF10. Notably, after DNA damage caused by oxaliplatin, the splicing factor SRSF10 controls a wide array of splicing decisions in the DNA damage response. These include the recognition of *BRCA1* exons 8–9 (legacy exons 9–10) and *CHEK2* exon 8, the latter of which undergoes AS-NMD as indicated above [43]. Taken together, these findings suggest that SRSF10 may be a key splicing factor implicated in the regulation of AS-NMD in BC susceptibility genes. Additional research into potential AS-NMD events in other BC genes is warranted as they may represent a suitable model for investigating their regulation.

The combined strategy consisting of bioinformatics analysis and minigene assays has identified a high proportion of spliceogenic variants (71%, 34/48 variants). Minigenes are valuable tools for studying splicing, offering insights for both basic and translational research. Among their key advantages, it is worthy to mention that minigenes allow the isolated study of mutant alleles without the interference of the wt allele that occurs in patient samples. Although direct patient RNA analysis is recommended for assessing potential spliceogenic variants, this sample may not always be readily available. Further, the simultaneous analysis of both wt and mutant alleles can entail a complex result interpretation, as the generation of full-length transcript by the mutant allele may be difficult to discern. Therefore, an integrated minigene/patient RNA strategy may be the most suitable approach. Also, minigenes demonstrate high versatility, as a single multi-exon minigene can be employed to analyze multiple variants, like mgATM_4-9 with 6 exons where 64 variants have been tested so far [28]. On the other hand, a main limitation is that minigenes are artificial constructs with incomplete genomic context, as they usually contain shortened introns. This can potentially lead to a lack of reproducibility in splicing outcomes when compared to patient RNA. However, it should be noted that this minigene was shown to replicate previous experimental carrier RNA results of four *ATM* variants (c.901 + 3 A > T; c.902-1G > T; 496 + 5G > A; c.1066-6 T > G), although it also revealed other minor transcripts due to the high sensitivity of our method [28, 51–54]. Further, we previously showed that minigene assays mimicked both physiological and variant-induced splicing patterns in more than 60 variants in different BC susceptibility genes where patient results were available [22, 28, 29, 55–60]. In this regard, it is critical to preserve the native genomic context in the minigene insert as much as possible, ensuring that the exon under investigation is flanked by its natural neighboring exons. In conclusion, mgATM_4-9 can be deemed as an appropriate tool for assessing variant spliceogenicity and conducting regulatory studies.

While in silico predictions of splice site sequences with MaxEntScan or SpliceAI are highly accurate [34, 61], the identification of active regulatory elements, such as splicing enhancers and silencers, has limitations as it shows high inaccuracy due to the enormous complexity of the splicing process [30, 34, 61–63]. Effective exon recognition depends on the interplay of numerous SRE motifs and their binding *trans*-acting factors so that point mutations may induce variable and context-dependent splicing anomalies [25]. Thus, in this study, depending on the specific variant tested, we have observed different levels of △(E7)-containing transcripts, ranging from 50% (c.876G > T) to 96.1% (c.680C > T) of the overall expression. Splicing outcomes cannot be reliably anticipated by any current algorithm, requiring functional evaluation as the most effective strategy for characterizing variant-induced impacts. HEXplorer predicted two putative ESE regions (c.665–681 and c.867–898) that were functionally corroborated in mgATM_4-9 [30]. Furthermore, the △HZEI score < −40 proved highly effective in identifying candidate variants, as △(E7)-transcripts were found in 34 out of 48 variants, with 19 exhibiting strong effects (mgFL-transcript < 30%). On the other hand, SpliceAI failed to predict any of the 34 spliceogenic variants detected in exon 7 (Supplementary Table S3).

Curiously, nine out of the nineteen most spliceogenic variants tested in this study are mapped in the interval c.665–681 (c.667G > C, c.667G > T, c.668A > T, c.669A > T, c.670A > T, c.672G > T, c.677C > T, c.680C > A, c.680C > T), confirming that it contains critical regulatory sequences. The remaining ten ESE-variants that exhibited higher levels of △(E7)-transcripts and mgFL-transcript levels < 30% are in the region c.867–898. Altogether, our data suggest that both segments, c.665–681 and c.867–898 are local clusters that actively regulate exon 7 identification and represent hotspots where a single nucleotide change may cause splicing anomalies. Taken together, this combination of in silico plus functional microdeletion data outperforms any SRE-prediction algorithm.

We previously provided evidence for a minigene-based haplosufficiency model, predicting that variants producing ≥ 30% of FL-transcripts are benign, whereas those generating ≤ 13% of FL-transcripts are pathogenic (provided that the remaining 87% expression meets the conditions for ATM loss-of-function). Variants in the 13–30% range have an undetermined effect on gene function and cancer risk [28]. According to the American College of Medical Genetics and Genomics/Association for Molecular Pathology (ACMG/AMP) guidelines and the specific recommendations for *ATM* [64–66], eight out of the 19 more spliceogenic variants would be classified as likely pathogenic (Supplementary Table S5). Thus, variants c.668A > T, c.680C > A and c.680C > T, which generate 12.9%, 5.5% and 3.9%, respectively, of mgFL-transcripts, were classified as likely pathogenic following this conservative model. Two of them are predicted missense changes (c.668A > T/p.(Glu223Val) and c.680C > T/p.(Ser227Leu)), underscoring that any alteration in the DNA sequence, regardless their predicted protein coding impact, could have a harmful impact on splicing. Moreover, six LP nonsense variants, which generate mgFL-transcripts with termination codons, would result in ATM loss-of-function through a double mechanism: splicing disruption and protein truncation. Up to 11 variants presented a significant reduction in mgFL-transcripts that was not sufficient for PVS1_(RNA) annotation, as mgFL levels were within the previously stablished 13–30% undetermined range. These changes would remain catalogued as VUS pending the precise establishment of the haploinsufficiency expression threshold for ATM. Although difficult to prove, it is possible that some of these variants are indeed *ATM* intermediate-risk alleles with reduced penetrance, or contribute to a complex hybrid mechanism of pathogenicity that impairs both protein function (e.g. missense variants) and splicing.

## Conclusions

These results demonstrate the critical need to incorporate splicing data when classifying exonic variants in alternatively spliced exons, regardless of their predicted impact on protein (missense, nonsense or synonymous). HEXplorer has provided valuable insights into potential enhancer or silencer regions within an exon, however, it is essential to experimentally validate any in silico prediction. Minigenes have once again demonstrated their potential for regulatory studies and high-throughput variant analysis. Hence, functional mapping by microdeletions in minigenes has proven to be a valuable approach for identifying SREs regulating atypical GC-5’ss, as well as non-constitutive exons as demonstrated by studies on *CHEK2* exons 8 and 10, *RAD51D* exon 3 and *ATM* exon 7, which are enriched in SREs [23–25]. This procedure allows to focus on specific regions to search for spliceogenic SRE-variants. Thus, so far, we have tested 177 variants located within the SRE-candidate intervals of these alternative exons, 102 of which (58%) disrupted their recognition. AS-NMD is a promising area for future research due to the thousands of such events in the human genome. A key direction is to systematically map and characterize the SREs that control these events. Identifying these elements and the variants that disrupt them are crucial for understanding disease mechanisms. For BC genes specifically, several common AS-NMD events are of particular interest and warrant further research. Examples include the *BRCA1* △(E8) and △(E9) transcripts, as well as the *ATM* △(E53) and △(E52_53) transcripts.

Our study has direct translational relevance at two key levels. First, it reclassifies certain *ATM* exon 7 variants—currently listed as VUS in ClinVar—as likely pathogenic. This reclassification enables more informed clinical decision-making for both carriers and their non-carrier relatives, turning previously ambiguous genetic findings into actionable insights, improving personalized risk assessment. Second, the study highlights *ATM* exon 7 as a hotspot for putative spliceogenic variants not detectable by conventional splicing prediction tools. Ultimately, this insight significantly contributes to refining the current ACMG/AMP guidelines for interpreting germline *ATM* sequence variants, underscoring the need for improved methodologies in variant analysis.

## Supplementary Information

Below is the link to the electronic supplementary material.

Supplementary file1 (PPTX 51 KB)

Supplementary file2 (PPTX 5.81 MB)

Supplementary file3 (PPTX 3.08 MB)

Supplementary file4 (DOCX 22 KB)

Supplementary file5 (DOCX 40 KB)

Supplementary file6 (DOCX 48.7 KB)

Supplementary file7 (DOCX 25 KB)

Supplementary file8 (DOCX 27 KB)

## Data Availability

All sequencing and fragment analysis data will be available in the DIGITAL.CSIC repository: http://hdl.handle.net/10261/377753 (10.20350/digitalCSIC/17026).

## References

[1] Paull TT (2015) Mechanisms of ATM activation. Annu Rev Biochem 84:711–738 25580527 10.1146/annurev-biochem-060614-034335

[2] Derheimer FA, Kastan MB (2010) Multiple roles of ATM in monitoring and maintaining DNA integrity. FEBS Lett 584:3675–3681 20580718 10.1016/j.febslet.2010.05.031 PMC2950315

[3] Thu KL, Yoon J-Y (2024) ATM-the gene at the moment in non-small cell lung cancer. Transl Lung Cancer Res 13:699–705 38601449 10.21037/tlcr-23-853 PMC11002499

[4] Savitsky K, Bar-Shira A, Gilad S et al (1995) A single ataxia telangiectasia gene with a product similar to PI-3 kinase. Science 268:1749–1753 7792600 10.1126/science.7792600

[5] Lesueur F, Easton DF, Renault A-L et al (2022) First international workshop of the ATM and cancer risk group (4–5 december 2019). Fam Cancer 21:211–227 34125377 10.1007/s10689-021-00248-y PMC9969796

[6] Hsu FC, Roberts NJ, Childs E et al (2021) Risk of pancreatic cancer among individuals with pathogenic variants in the ATM gene. JAMA Oncol 7:1664–1668 34529012 10.1001/jamaoncol.2021.3701 PMC8446906

[7] Narod SA (2021) Which genes for hereditary breast cancer? N Engl J Med 384:471–473 33471975 10.1056/NEJMe2035083

[8] Dorling L, Carvalho S, Allen J et al (2021) Breast cancer risk genes — association analysis in more than 113,000 women. N Engl J Med 384:428–439 33471991 10.1056/NEJMoa1913948 PMC7611105

[9] Hu C, Hart SN, Gnanaolivu R et al (2021) A population-based study of genes previously implicated in breast cancer. N Engl J Med 384:440–451 33471974 10.1056/NEJMoa2005936 PMC8127622

[10] Foulkes WD (2021) The ten genes for breast (and ovarian) cancer susceptibility. Nat Rev Clin Oncol 18:259–260 33692540 10.1038/s41571-021-00491-3

[11] Yadav S, LaDuca H, Polley EC et al (2020) Racial and ethnic differences in multigene hereditary cancer panel test results for women with breast cancer. JNCI J Natl Cancer Inst 113:1429–1433 10.1093/jnci/djaa167 PMC8633452 33146377

[12] Lowry KP, Geuzinge HA, Stout NK et al (2022) Breast cancer screening strategies for women with ATM, CHEK2, and PALB2 pathogenic variants: a comparative modeling analysis. JAMA Oncol 8:587–596 35175286 10.1001/jamaoncol.2021.6204 PMC8855312

[13] Marasco LE, Kornblihtt AR (2023) The physiology of alternative splicing. Nat Rev Mol Cell Biol 24:242–254 36229538 10.1038/s41580-022-00545-z

[14] Manning KS, Cooper TA (2017) The roles of RNA processing in translating genotype to phenotype. Nat Rev Mol Cell Biol 18:102–114 27847391 10.1038/nrm.2016.139 PMC5544131

[15] Rogalska ME, Vivori C, Valcárcel J (2023) Regulation of pre-mRNA splicing: roles in physiology and disease, and therapeutic prospects. Nat Rev Genet 24:251–269 36526860 10.1038/s41576-022-00556-8

[16] Cartegni L, Chew SL, Krainer AR (2002) Listening to silence and understanding nonsense: exonic mutations that affect splicing. Nat Rev Genet 3:285–298 11967553 10.1038/nrg775

[17] Lopez-Bigas N, Audit B, Ouzounis C et al (2005) Are splicing mutations the most frequent cause of hereditary disease? FEBS Lett 579:1900–1903 15792793 10.1016/j.febslet.2005.02.047

[18] Rhine CL, Cygan KJ, Soemedi R et al (2018) Hereditary cancer genes are highly susceptible to splicing mutations. PLoS Genet 14:e1007231 29505604 10.1371/journal.pgen.1007231 PMC5854443

[19] Sanz DJ, Acedo A, Infante M et al (2010) A high proportion of DNA variants of BRCA1 and BRCA2 is associated with aberrant splicing in breast/ovarian cancer patients. Clin Cancer Res 16:1957–1967 20215541 10.1158/1078-0432.CCR-09-2564

[20] Baralle D, Lucassen A, Buratti E (2009) Missed threads. The impact of pre-mRNA splicing defects on clinical practice. EMBO Rep 10:810–816 19648957 10.1038/embor.2009.170 PMC2726684

[21] Singh G, Cooper TA (2006) Minigene reporter for identification and analysis of *cis* elements and *trans* factors affecting pre-mRNA splicing. Biotechniques 41:177–181 16925019 10.2144/000112208

[22] Acedo A, Hernández-Moro C, Curiel-García Á et al (2015) Functional classification of BRCA2 DNA variants by splicing assays in a large minigene with 9 exons. Hum Mutat 36:210–221 25382762 10.1002/humu.22725 PMC4371643

[23] Llinares-Burguet I, Sanoguera-Miralles L, Valenzuela-Palomo A et al (2024) Splicing dysregulation of non-canonical GC-5′ splice sites of breast cancer susceptibility genes ATM and PALB2. Cancers 16:3562 39518003 10.3390/cancers16213562 PMC11545216

[24] Bueno-Martínez E, Sanoguera-Miralles L, Valenzuela-Palomo A et al (2021) RAD51D aberrant splicing in breast cancer: identification of splicing regulatory elements and minigene-based evaluation of 53 DNA variants. Cancers 13:2845 34200360 10.3390/cancers13112845 PMC8201001

[25] Sanoguera-Miralles L, Llinares-Burguet I, Bueno-Martínez E et al (2024) Comprehensive splicing analysis of the alternatively spliced CHEK2 exons 8 and 10 reveals three enhancer/silencer-rich regions and 38 spliceogenic variants. J Pathol 262:395–409 38332730 10.1002/path.6243

[26] Dawes R, Bournazos AM, Bryen SJ et al (2023) Splicevault predicts the precise nature of variant-associated mis-splicing. Nat Genet 55:324–332 36747048 10.1038/s41588-022-01293-8 PMC9925382

[27] Fair B, Najar CFBA, Zhao J et al (2024) Global impact of unproductive splicing on human gene expression. Nat Genet 56:1851–1861 39223315 10.1038/s41588-024-01872-x PMC11387194

[28] Bueno-Martínez E, Sanoguera-Miralles L, Valenzuela-Palomo A et al (2022) Minigene-based splicing analysis and ACMG/AMP-based tentative classification of 56 ATM variants. J Pathol 258:83–101 35716007 10.1002/path.5979 PMC9541484

[29] Fraile-Bethencourt E, Valenzuela-Palomo A, Díez-Gómez B et al (2019) Minigene splicing assays identify 12 spliceogenic variants of BRCA2 exons 14 and 15. Front Genet 10:503 31191615 10.3389/fgene.2019.00503 PMC6546720

[30] Erkelenz S, Theiss S, Otte M et al (2014) Genomic HEXploring allows landscaping of novel potential splicing regulatory elements. Nucleic Acids Res 42:10681–10697 25147205 10.1093/nar/gku736 PMC4176321

[31] Canson D, Glubb D, Spurdle AB (2020) Variant effect on splicing regulatory elements, branchpoint usage, and pseudoexonization: strategies to enhance bioinformatic prediction using hereditary cancer genes as exemplars. Hum Mutat 41:1705–1721 32623769 10.1002/humu.24074

[32] Tubeuf H, Charbonnier C, Soukarieh O et al (2020) Large-scale comparative evaluation of user-friendly tools for predicting variant-induced alterations of splicing regulatory elements. Hum Mutat 41:1811–1829 32741062 10.1002/humu.24091

[33] Canson DM, Llinares-Burguet I, Fortuno C et al (2025) TP53 minigene analysis of 161 sequence changes provides evidence for role of spatial constraint and regulatory elements on variant-induced splicing impact. NPJ Genomic Med 10:37 10.1038/s41525-025-00498-0 PMC12062376 40341019

[34] Jaganathan K, Panagiotopoulou SK, McRae JF et al (2019) Predicting splicing from primary sequence with deep learning. Cell 176:535-548.e24 30661751 10.1016/j.cell.2018.12.015

[35] Grønning AGB, Doktor TK, Larsen SJ et al (2020) DeepCLIP: predicting the effect of mutations on protein-RNA binding with deep learning. Nucleic Acids Res 48:7099–7118 32558887 10.1093/nar/gkaa530 PMC7367176

[36] de Garibay GR, Acedo A, García-Casado Z et al (2014) Capillary electrophoresis analysis of conventional splicing assays: IARC analytical and clinical classification of 31 BRCA2 genetic variants. Hum Mutat 35:53–57 24123850 10.1002/humu.22456

[37] Gabriel M, Delforge Y, Deward A et al (2015) Role of the splicing factor SRSF4 in cisplatin-induced modifications of pre-mRNA splicing and apoptosis. BMC Cancer 15:227 25884497 10.1186/s12885-015-1259-0 PMC4399393

[38] Kadota Y, Jam FA, Yukiue H et al (2020) Srsf7 establishes the juvenile transcriptome through age-dependent alternative splicing in mice. iScience 23:100929 32146325 10.1016/j.isci.2020.100929 PMC7063262

[39] Kralovicova J, Hwang G, Asplund aC et al (2011) Compensatory signals associated with the activation of human GC 5’ splice sites. Nucleic Acids Res 39:7077–7091 21609956 10.1093/nar/gkr306 PMC3167603

[40] Erkelenz S, Mueller WF, Evans MS et al (2013) Position-dependent splicing activation and repression by SR and hnRNP proteins rely on common mechanisms. RNA 19:96–102 23175589 10.1261/rna.037044.112 PMC3527730

[41] Shkreta L, Delannoy A, Salvetti A, Chabot B (2021) SRSF10: an atypical splicing regulator with critical roles in stress response, organ development, and viral replication. RNA 27:1302–1317 34315816 10.1261/rna.078879.121 PMC8522700

[42] Shin C, Feng Y, Manley JL (2004) Dephosphorylated SRp38 acts as a splicing repressor in response to heat shock. Nature 427:553–558 14765198 10.1038/nature02288

[43] Shkreta L, Toutant J, Durand M et al (2016) Srsf10 connects DNA damage to the alternative splicing of transcripts encoding apoptosis, cell-cycle control, and DNA repair factors. Cell Rep 17:1990–2003 27851963 10.1016/j.celrep.2016.10.071 PMC5483951

[44] Corvelo A, Eyras E (2008) Exon creation and establishment in human genes. Genome Biol 9:R141 18811936 10.1186/gb-2008-9-9-r141 PMC2592719

[45] Hertel KJ (2008) Combinatorial control of exon recognition. J Biol Chem 283:1211–1215 18024426 10.1074/jbc.R700035200

[46] Sullivan PJ, Quinn JMW, Wu W et al (2024) SpliceVarDB: a comprehensive database of experimentally validated human splicing variants. Am J Hum Genet 111:2164–2175 39226898 10.1016/j.ajhg.2024.08.002 PMC11480807

[47] Krawczak M, Reiss J, Cooper DN (1992) The mutational spectrum of single base-pair substitutions in mRNA splice junctions of human genes: causes and consequences. Hum Genet 90:41–54 1427786 10.1007/BF00210743

[48] Buratti E, Baralle M, Baralle FE (2006) Defective splicing, disease and therapy: searching for master checkpoints in exon definition. Nucleic Acids Res 34:3494–3510 16855287 10.1093/nar/gkl498 PMC1524908

[49] Lareau LF, Inada M, Green RE et al (2007) Unproductive splicing of SR genes associated with highly conserved and ultraconserved DNA elements. Nature 446:926–929 17361132 10.1038/nature05676

[50] Blackford AN, Jackson SP (2017) ATM, ATR, and DNA-PK: the trinity at the heart of the DNA damage response. Mol Cell 66:801–817 28622525 10.1016/j.molcel.2017.05.015

[51] Laake K, Jansen L, Hahnemann JM et al (2000) Characterization of ATM mutations in 41 Nordic families with ataxia telangiectasia. Hum Mutat 16:232–246 10980530 10.1002/1098-1004(200009)16:3<232::AID-HUMU6>3.0.CO;2-L

[52] Teraoka SN, Telatar M, Becker-Catania S et al (1999) Splicing defects in the ataxia-telangiectasia gene, ATM: underlying mutations and consequences. Am J Hum Genet 64:1617–1631 10330348 10.1086/302418 PMC1377904

[53] Dörk T, Bendix-Waltes R, Wegner RD, Stumm M (2004) Slow progression of Ataxia-telangiectasia with double missense and in frame splice mutations. Am J Med Genet 126 A:272–277 10.1002/ajmg.a.20601 15054841

[54] Soukupova J, Dundr P, Kliebl Z, Pohlreich P (2008) Contribution of mutations in ATM to breast cancer development in the Czech population. Oncol Rep 19:1505–1510 18497957

[55] Fraile-Bethencourt E, Díez-Gómez B, Velásquez-Zapata V et al (2017) Functional classification of DNA variants by hybrid minigenes: identification of 30 spliceogenic variants of BRCA2 exons 17 and 18. PLoS Genet 13:e1006691 28339459 10.1371/journal.pgen.1006691 PMC5384790

[56] Fraile-Bethencourt E, Valenzuela-Palomo A, Díez-Gómez B et al (2018) Identification of eight spliceogenic variants in BRCA2 exon 16 by minigene assays. Front Genet 9:188 29881398 10.3389/fgene.2018.00188 PMC5977032

[57] Fraile-Bethencourt E, Valenzuela-Palomo A, Díez-Gómez B et al (2019) Mis-splicing in breast cancer: identification of pathogenic BRCA2 variants by systematic minigene assays. J Pathol 248:409–420 30883759 10.1002/path.5268

[58] Sanoguera-Miralles L, Valenzuela-Palomo A, Bueno-Martínez E et al (2020) Comprehensive functional characterization and clinical interpretation of 20 splice-site variants of the RAD51C gene. Cancers 12:3771 33333735 10.3390/cancers12123771 PMC7765170

[59] Valenzuela-Palomo A, Bueno-Martínez E, Sanoguera-Miralles L et al (2022) Splicing predictions, minigene analyses, and ACMG-AMP clinical classification of 42 germline PALB2 splice-site variants. J Pathol 256:321–334 34846068 10.1002/path.5839 PMC9306493

[60] Sanoguera-Miralles L, Valenzuela-Palomo A, Bueno-Martínez E et al (2024) Systematic minigene-based splicing analysis and tentative clinical classification of 52 CHEK2 splice-site variants. Clin Chem 70:319–338 37725924 10.1093/clinchem/hvad125

[61] Yeo G, Burge CB (2004) Maximum entropy modeling of short sequence motifs with applications to RNA splicing signals. J Comput Biol 11:377–394 15285897 10.1089/1066527041410418

[62] Cartegni L, Wang J, Zhu Z et al (2003) ESEfinder: a web resource to identify exonic splicing enhancers. Nucleic Acids Res 31:3568–3571 12824367 10.1093/nar/gkg616 PMC169022

[63] Piva F, Giulietti M, Nocchi L, Principato G (2009) Spliceaid: a database of experimental RNA target motifs bound by splicing proteins in humans. Bioinformatics 25:1211–1213 19261717 10.1093/bioinformatics/btp124

[64] Richards S, Aziz N, Bale S et al (2015) Standards and guidelines for the interpretation of sequence variants: a joint consensus recommendation of the American College of Medical Genetics and Genomics and the Association for Molecular Pathology. Genet Med 17:405–424 25741868 10.1038/gim.2015.30 PMC4544753

[65] Feliubadaló L, Moles-Fernández A, Santamariña-Pena M et al (2021) A collaborative effort to define classification criteria for ATM variants in hereditary cancer patients. Clin Chem 67:518–533 33280026 10.1093/clinchem/hvaa250

[66] Richardson ME, Holdren M, Brannan T et al (2024) Specifications of the ACMG/AMP variant curation guidelines for the analysis of germline ATM sequence variants. Am J Hum Genet 111:2411–2426 39317201 10.1016/j.ajhg.2024.08.022 PMC11568761

