# Supplementary material for: Unproductive alternative splicing of ATM exon 7: mapping of critical regulatory elements and identification of 34 spliceogenic variants
Source: J Mol Med (Berl). 2025 Sep 20;103(11-12):1447–60. doi: 10.1007/s00109-025-02595-0 (PMC12675606; doi:10.1007/s00109-025-02595-0)
Supplement: Supplementary file 1 — Supplementary file1 (PPTX 51 KB) [file 109_2025_2595_MOESM1_ESM.pptx]

## Slide 1
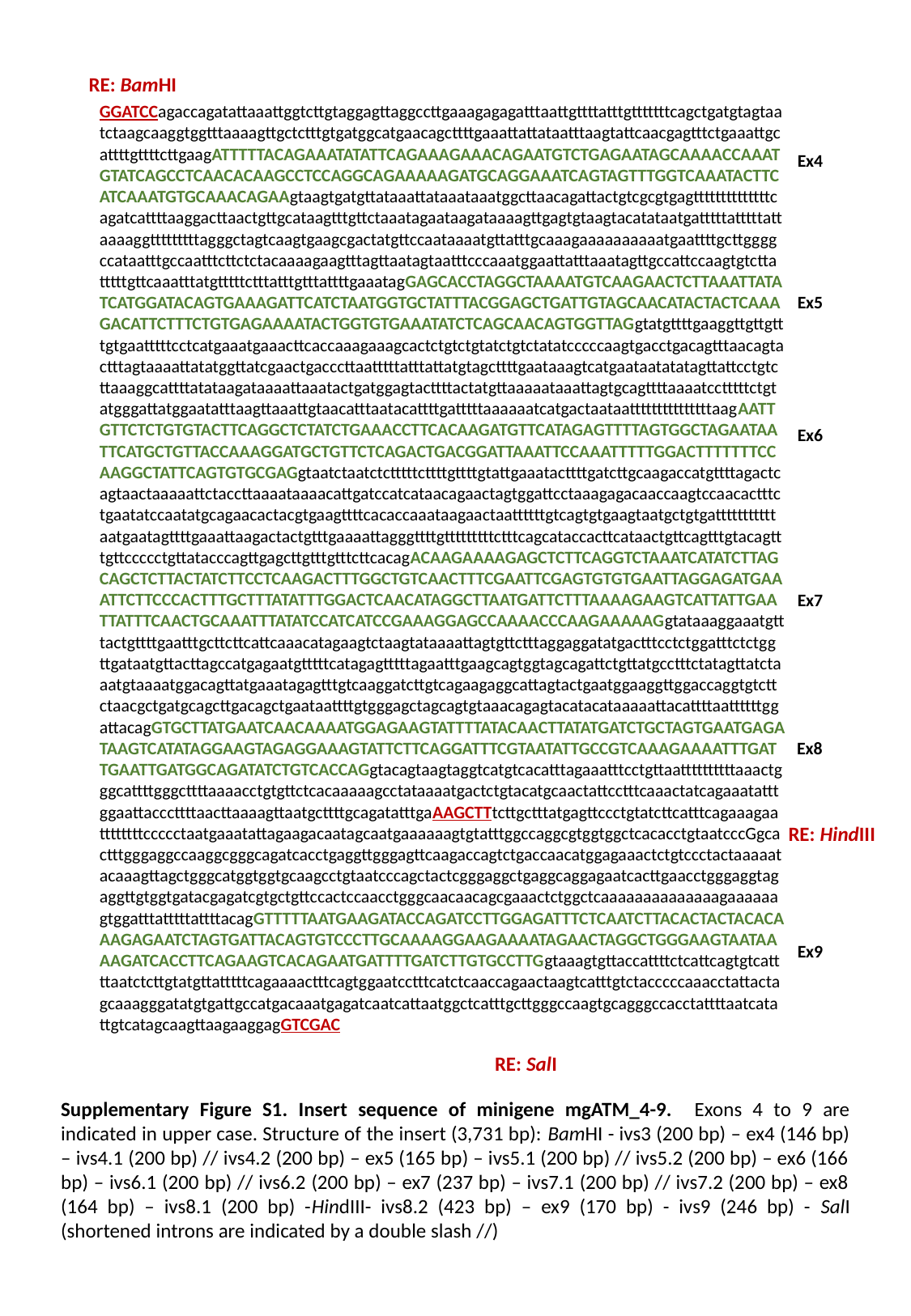

RE: BamHI
GGATCCagaccagatattaaattggtcttgtaggagttaggccttgaaagagagatttaattgttttatttgtttttttcagctgatgtagtaatctaagcaaggtggtttaaaagttgctctttgtgatggcatgaacagcttttgaaattattataatttaagtattcaacgagtttctgaaattgcattttgttttcttgaagATTTTTACAGAAATATATTCAGAAAGAAACAGAATGTCTGAGAATAGCAAAACCAAATGTATCAGCCTCAACACAAGCCTCCAGGCAGAAAAAGATGCAGGAAATCAGTAGTTTGGTCAAATACTTCATCAAATGTGCAAACAGAAgtaagtgatgttataaattataaataaatggcttaacagattactgtcgcgtgagttttttttttttttcagatcattttaaggacttaactgttgcataagtttgttctaaatagaataagataaaagttgagtgtaagtacatataatgatttttatttttattaaaaggtttttttttagggctagtcaagtgaagcgactatgttccaataaaatgttatttgcaaagaaaaaaaaaatgaattttgcttggggccataatttgccaatttcttctctacaaaagaagtttagttaatagtaatttcccaaatggaattatttaaatagttgccattccaagtgtcttatttttgttcaaatttatgtttttctttatttgtttattttgaaatagGAGCACCTAGGCTAAAATGTCAAGAACTCTTAAATTATATCATGGATACAGTGAAAGATTCATCTAATGGTGCTATTTACGGAGCTGATTGTAGCAACATACTACTCAAAGACATTCTTTCTGTGAGAAAATACTGGTGTGAAATATCTCAGCAACAGTGGTTAGgtatgttttgaaggttgttgtttgtgaatttttcctcatgaaatgaaacttcaccaaagaaagcactctgtctgtatctgtctatatcccccaagtgacctgacagtttaacagtactttagtaaaattatatggttatcgaactgacccttaatttttatttattatgtagcttttgaataaagtcatgaataatatatagttattcctgtcttaaaggcattttatataagataaaattaaatactgatggagtacttttactatgttaaaaataaattagtgcagttttaaaatcctttttctgtatgggattatggaatatttaagttaaattgtaacatttaatacattttgatttttaaaaaatcatgactaataatttttttttttttttaagAATTGTTCTCTGTGTACTTCAGGCTCTATCTGAAACCTTCACAAGATGTTCATAGAGTTTTAGTGGCTAGAATAATTCATGCTGTTACCAAAGGATGCTGTTCTCAGACTGACGGATTAAATTCCAAATTTTTGGACTTTTTTTCCAAGGCTATTCAGTGTGCGAGgtaatctaatctctttttcttttgttttgtattgaaatacttttgatcttgcaagaccatgttttagactcagtaactaaaaattctaccttaaaataaaacattgatccatcataacagaactagtggattcctaaagagacaaccaagtccaacactttctgaatatccaatatgcagaacactacgtgaagttttcacaccaaataagaactaattttttgtcagtgtgaagtaatgctgtgatttttttttttaatgaatagttttgaaattaagactactgtttgaaaattagggttttgtttttttttctttcagcataccacttcataactgttcagtttgtacagtttgttccccctgttatacccagttgagcttgtttgtttcttcacagACAAGAAAAGAGCTCTTCAGGTCTAAATCATATCTTAGCAGCTCTTACTATCTTCCTCAAGACTTTGGCTGTCAACTTTCGAATTCGAGTGTGTGAATTAGGAGATGAAATTCTTCCCACTTTGCTTTATATTTGGACTCAACATAGGCTTAATGATTCTTTAAAAGAAGTCATTATTGAATTATTTCAACTGCAAATTTATATCCATCATCCGAAAGGAGCCAAAACCCAAGAAAAAGgtataaaggaaatgtttactgttttgaatttgcttcttcattcaaacatagaagtctaagtataaaattagtgttctttaggaggatatgactttcctctggatttctctggttgataatgttacttagccatgagaatgtttttcatagagtttttagaatttgaagcagtggtagcagattctgttatgcctttctatagttatctaaatgtaaaatggacagttatgaaatagagtttgtcaaggatcttgtcagaagaggcattagtactgaatggaaggttggaccaggtgtcttctaacgctgatgcagcttgacagctgaataattttgtgggagctagcagtgtaaacagagtacatacataaaaattacattttaattttttggattacagGTGCTTATGAATCAACAAAATGGAGAAGTATTTTATACAACTTATATGATCTGCTAGTGAATGAGATAAGTCATATAGGAAGTAGAGGAAAGTATTCTTCAGGATTTCGTAATATTGCCGTCAAAGAAAATTTGATTGAATTGATGGCAGATATCTGTCACCAGgtacagtaagtaggtcatgtcacatttagaaatttcctgttaattttttttttaaactgggcattttgggcttttaaaacctgtgttctcacaaaaagcctataaaatgactctgtacatgcaactattcctttcaaactatcagaaatatttggaattacccttttaacttaaaagttaatgcttttgcagatatttgaAAGCTTtcttgctttatgagttccctgtatcttcatttcagaaagaattttttttccccctaatgaaatattagaagacaatagcaatgaaaaaagtgtatttggccaggcgtggtggctcacacctgtaatcccGgcactttgggaggccaaggcgggcagatcacctgaggttgggagttcaagaccagtctgaccaacatggagaaactctgtccctactaaaaatacaaagttagctgggcatggtggtgcaagcctgtaatcccagctactcgggaggctgaggcaggagaatcacttgaacctgggaggtagaggttgtggtgatacgagatcgtgctgttccactccaacctgggcaacaacagcgaaactctggctcaaaaaaaaaaaaaagaaaaaagtggatttatttttattttacagGTTTTTAATGAAGATACCAGATCCTTGGAGATTTCTCAATCTTACACTACTACACAAAGAGAATCTAGTGATTACAGTGTCCCTTGCAAAAGGAAGAAAATAGAACTAGGCTGGGAAGTAATAAAAGATCACCTTCAGAAGTCACAGAATGATTTTGATCTTGTGCCTTGgtaaagtgttaccattttctcattcagtgtcattttaatctcttgtatgttatttttcagaaaactttcagtggaatcctttcatctcaaccagaactaagtcatttgtctacccccaaacctattactagcaaagggatatgtgattgccatgacaaatgagatcaatcattaatggctcatttgcttgggccaagtgcagggccacctattttaatcatattgtcatagcaagttaagaaggagGTCGAC
Ex4
Ex5
Ex6
Ex7
Ex8
Ex9
RE: HindIII
RE: SalI
Supplementary Figure S1. Insert sequence of minigene mgATM_4-9. Exons 4 to 9 are indicated in upper case. Structure of the insert (3,731 bp): BamHI - ivs3 (200 bp) – ex4 (146 bp) – ivs4.1 (200 bp) // ivs4.2 (200 bp) – ex5 (165 bp) – ivs5.1 (200 bp) // ivs5.2 (200 bp) – ex6 (166 bp) – ivs6.1 (200 bp) // ivs6.2 (200 bp) – ex7 (237 bp) – ivs7.1 (200 bp) // ivs7.2 (200 bp) – ex8 (164 bp) – ivs8.1 (200 bp) -HindIII- ivs8.2 (423 bp) – ex9 (170 bp) - ivs9 (246 bp) - SalI (shortened introns are indicated by a double slash //)
