# Supplementary material for: Unproductive alternative splicing of ATM exon 7: mapping of critical regulatory elements and identification of 34 spliceogenic variants
Source: J Mol Med (Berl). 2025 Sep 20;103(11-12):1447–60. doi: 10.1007/s00109-025-02595-0 (PMC12675606; doi:10.1007/s00109-025-02595-0)
Supplement: Supplementary file 2 — Supplementary file2 (PPTX 5.81 MB) [file 109_2025_2595_MOESM2_ESM.pptx]

## Slide 1
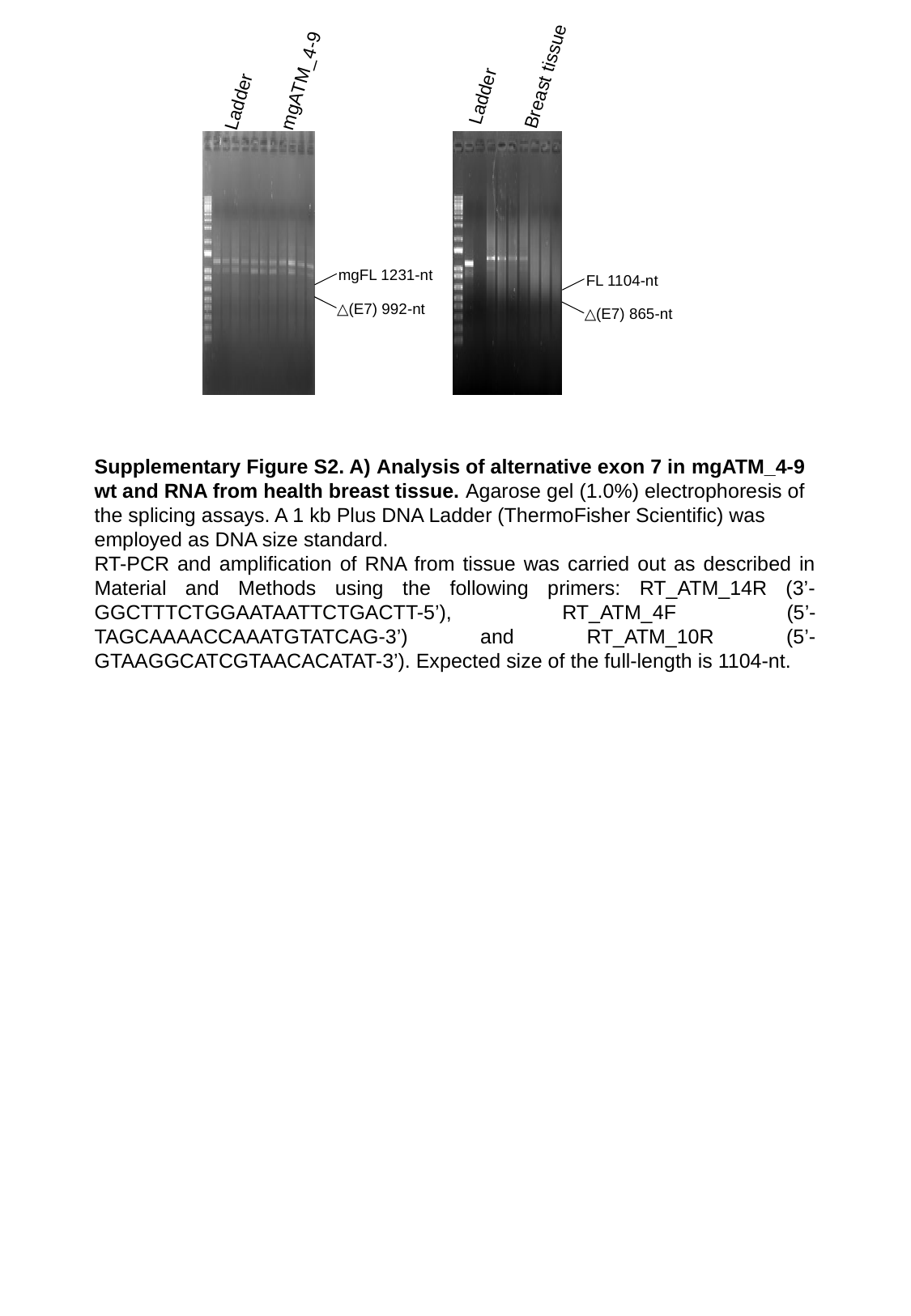

Breast tissue
Ladder
mgATM_4-9
Ladder
mgFL 1231-nt
△(E7) 992-nt
FL 1104-nt
△(E7) 865-nt
Supplementary Figure S2. A) Analysis of alternative exon 7 in mgATM_4-9 wt and RNA from health breast tissue. Agarose gel (1.0%) electrophoresis of the splicing assays. A 1 kb Plus DNA Ladder (ThermoFisher Scientific) was employed as DNA size standard.
RT-PCR and amplification of RNA from tissue was carried out as described in Material and Methods using the following primers: RT_ATM_14R (3’- GGCTTTCTGGAATAATTCTGACTT-5’), RT_ATM_4F (5’- TAGCAAAACCAAATGTATCAG-3’) and RT_ATM_10R (5’- GTAAGGCATCGTAACACATAT-3’). Expected size of the full-length is 1104-nt.

## Slide 2
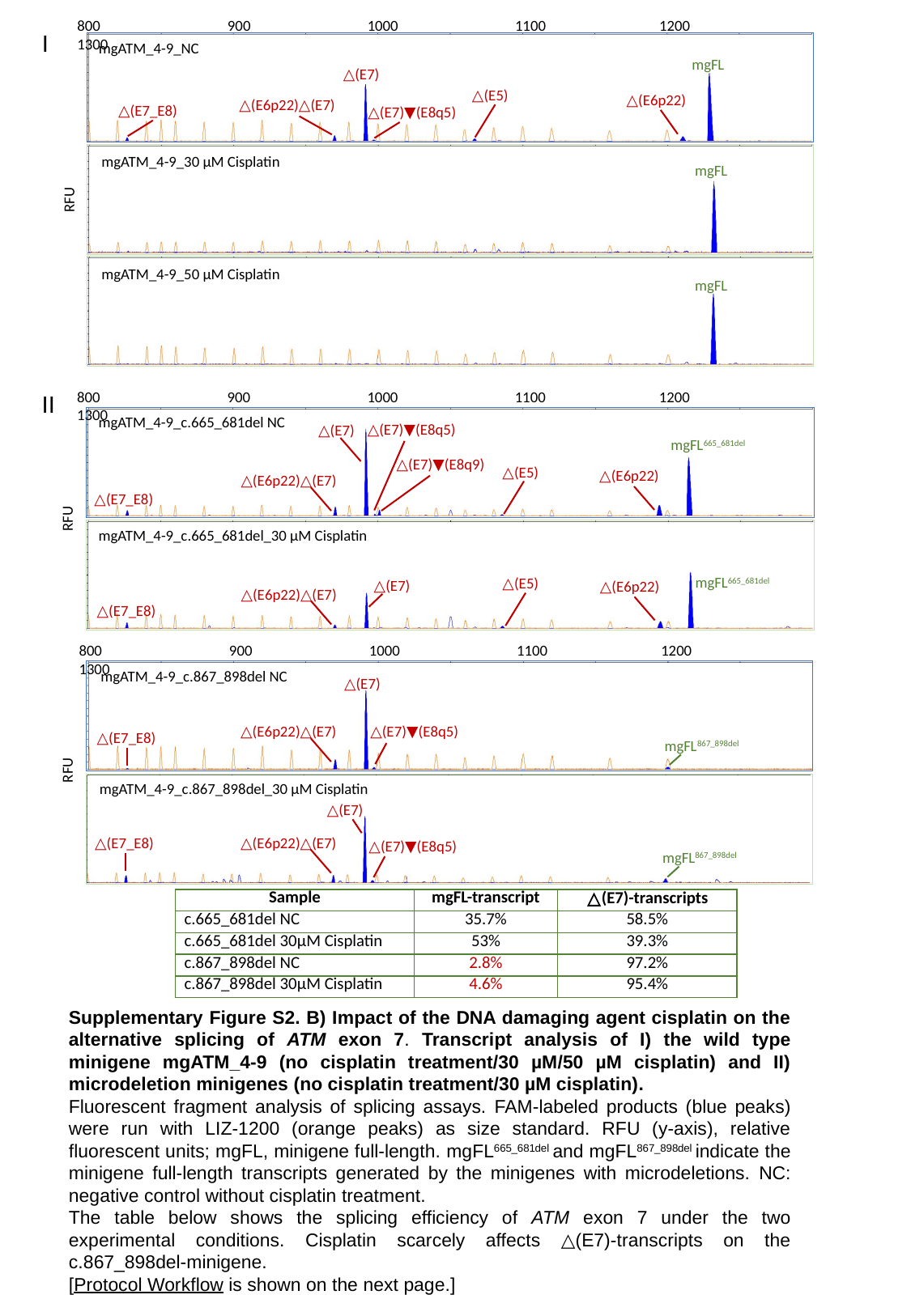

800 900 1000 1100 1200 1300
RFU
mgATM_4-9_NC
mgFL
△(E7)
△(E5)
△(E6p22)
△(E6p22)△(E7)
△(E7_E8)
△(E7)▼(E8q5)
mgATM_4-9_30 µM Cisplatin
mgFL
mgATM_4-9_50 µM Cisplatin
mgFL
I
800 900 1000 1100 1200 1300
mgATM_4-9_c.665_681del NC
RFU
△(E7)▼(E8q5)
△(E7)
mgFL665_681del
△(E7)▼(E8q9)
△(E6p22)
△(E6p22)△(E7)
△(E7_E8)
mgATM_4-9_c.665_681del_30 µM Cisplatin
mgFL665_681del
△(E7)
△(E6p22)
△(E6p22)△(E7)
△(E7_E8)
800 900 1000 1100 1200 1300
RFU
mgATM_4-9_c.867_898del NC
△(E7)
△(E6p22)△(E7)
△(E7)▼(E8q5)
△(E7_E8)
mgFL867_898del
mgATM_4-9_c.867_898del_30 µM Cisplatin
△(E7)
△(E7_E8)
△(E6p22)△(E7)
△(E7)▼(E8q5)
mgFL867_898del
II
△(E5)
△(E5)
| Sample | mgFL-transcript | △(E7)-transcripts |
| --- | --- | --- |
| c.665\_681del NC | 35.7% | 58.5% |
| c.665\_681del 30µM Cisplatin | 53% | 39.3% |
| c.867\_898del NC | 2.8% | 97.2% |
| c.867\_898del 30µM Cisplatin | 4.6% | 95.4% |
Supplementary Figure S2. B) Impact of the DNA damaging agent cisplatin on the alternative splicing of ATM exon 7. Transcript analysis of I) the wild type minigene mgATM_4-9 (no cisplatin treatment/30 µM/50 µM cisplatin) and II) microdeletion minigenes (no cisplatin treatment/30 µM cisplatin).
Fluorescent fragment analysis of splicing assays. FAM-labeled products (blue peaks) were run with LIZ-1200 (orange peaks) as size standard. RFU (y-axis), relative fluorescent units; mgFL, minigene full-length. mgFL665_681del and mgFL867_898del indicate the minigene full-length transcripts generated by the minigenes with microdeletions. NC: negative control without cisplatin treatment.
The table below shows the splicing efficiency of ATM exon 7 under the two experimental conditions. Cisplatin scarcely affects △(E7)-transcripts on the c.867_898del-minigene.
[Protocol Workflow is shown on the next page.]

## Slide 3
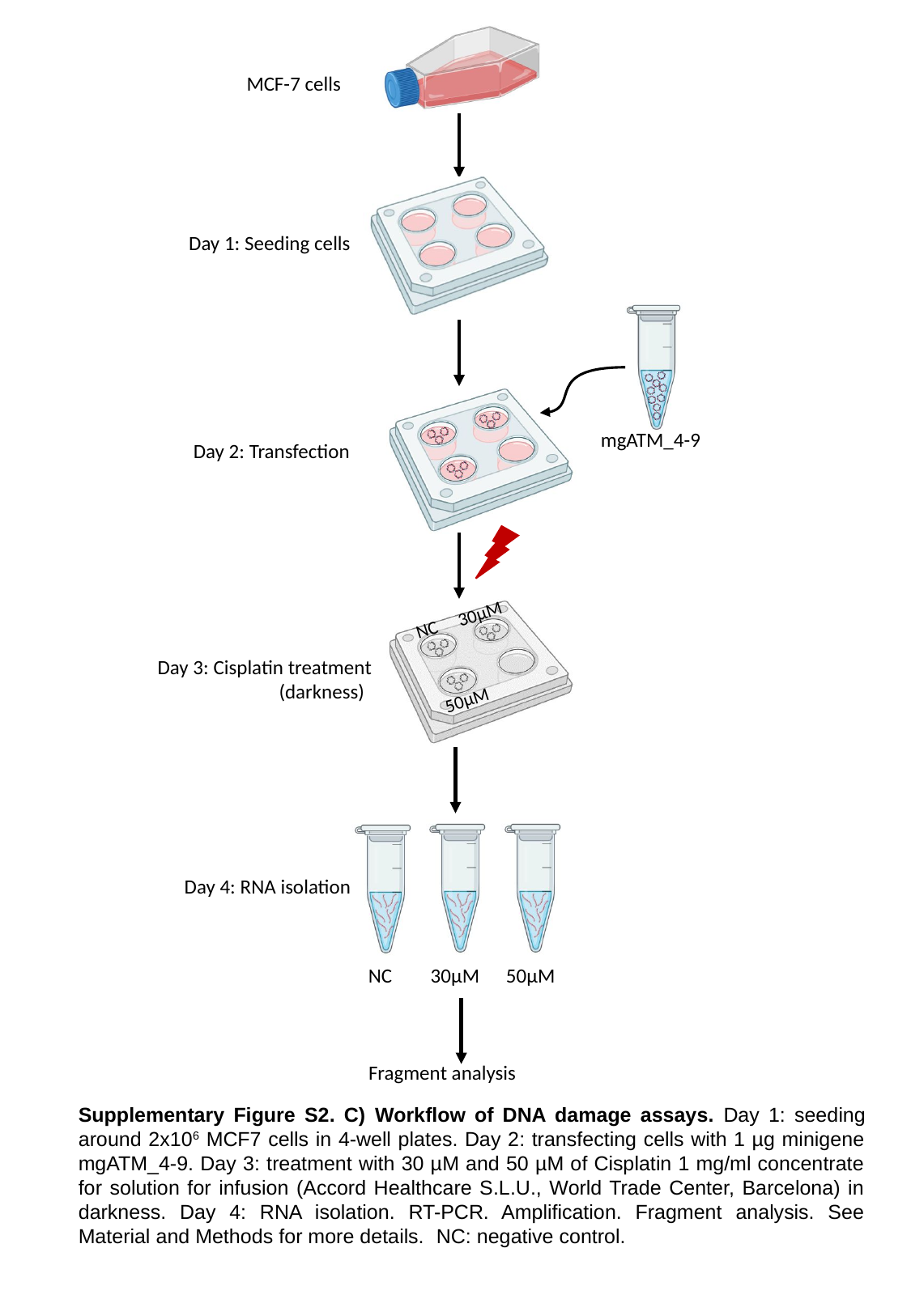

MCF-7 cells
Day 1: Seeding cells
mgATM_4-9
Day 2: Transfection
30µM
NC
Day 3: Cisplatin treatment
	(darkness)
50µM
Day 4: RNA isolation
NC
30µM
50µM
Fragment analysis
Supplementary Figure S2. C) Workflow of DNA damage assays. Day 1: seeding around 2x10^6^ MCF7 cells in 4-well plates. Day 2: transfecting cells with 1 µg minigene mgATM_4-9. Day 3: treatment with 30 µM and 50 µM of Cisplatin 1 mg/ml concentrate for solution for infusion (Accord Healthcare S.L.U., World Trade Center, Barcelona) in darkness. Day 4: RNA isolation. RT-PCR. Amplification. Fragment analysis. See Material and Methods for more details. NC: negative control.
