# Supplementary material for: Unproductive alternative splicing of ATM exon 7: mapping of critical regulatory elements and identification of 34 spliceogenic variants
Source: J Mol Med (Berl). 2025 Sep 20;103(11-12):1447–60. doi: 10.1007/s00109-025-02595-0 (PMC12675606; doi:10.1007/s00109-025-02595-0)
Supplement: Supplementary file 3 — Supplementary file3 (PPTX 3.08 MB) [file 109_2025_2595_MOESM3_ESM.pptx]

## Slide 1
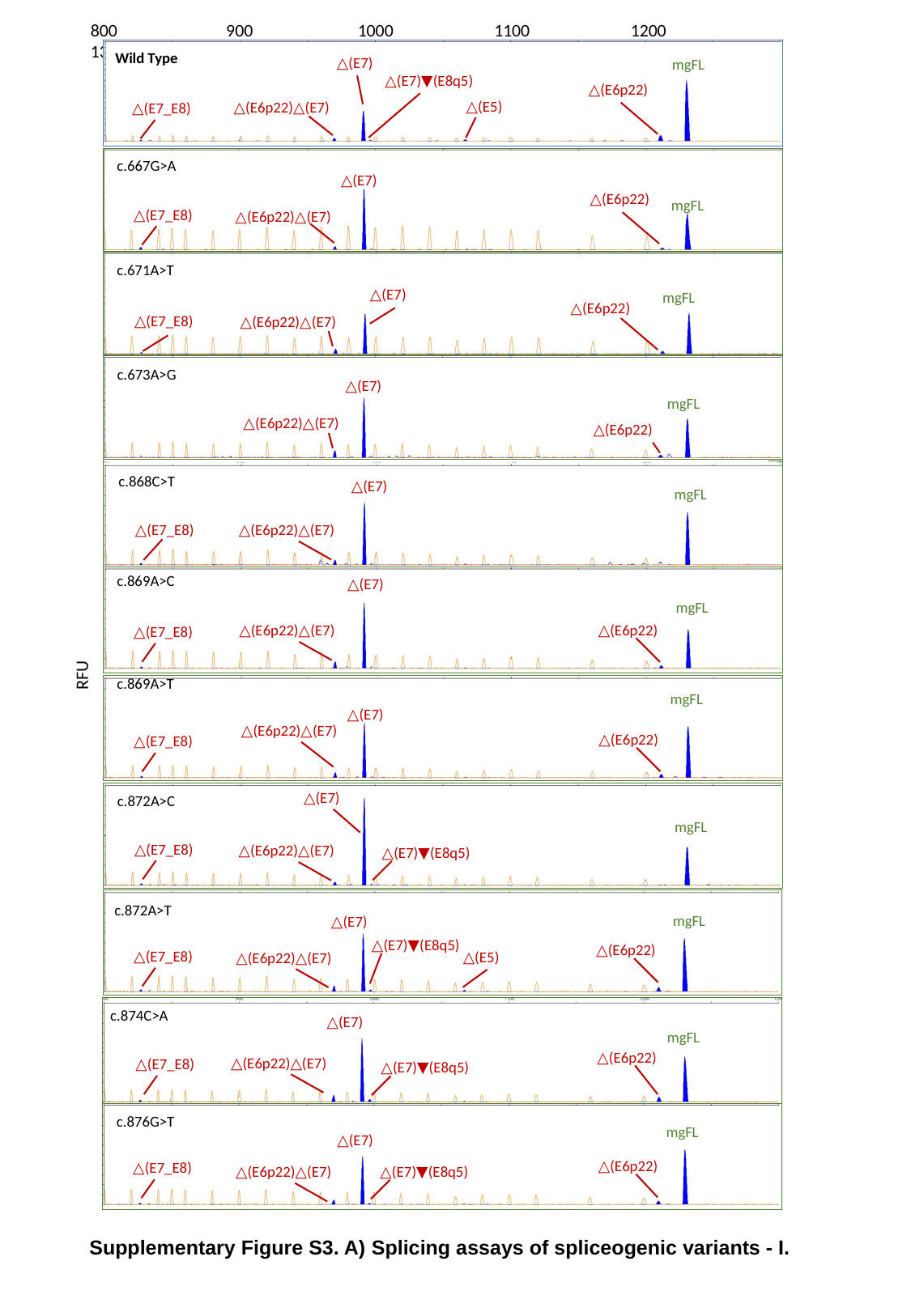

800 900 1000 1100 1200 1300
Wild Type
△(E7)
mgFL
△(E7)▼(E8q5)
△(E6p22)
△(E5)
△(E6p22)△(E7)
△(E7_E8)
c.667G>A
△(E7)
△(E6p22)
mgFL
△(E7_E8)
△(E6p22)△(E7)
c.671A>T
△(E7)
mgFL
△(E6p22)
△(E7_E8)
△(E6p22)△(E7)
c.673A>G
△(E7)
mgFL
△(E6p22)△(E7)
△(E6p22)
c.868C>T
△(E7)
mgFL
△(E7_E8)
△(E6p22)△(E7)
c.869A>C
△(E7)
mgFL
△(E6p22)
△(E6p22)△(E7)
△(E7_E8)
c.869A>T
mgFL
△(E7)
△(E6p22)△(E7)
RFU
△(E6p22)
△(E7_E8)
△(E7)
c.872A>C
mgFL
△(E6p22)△(E7)
△(E7_E8)
△(E7)▼(E8q5)
c.872A>T
mgFL
△(E7)
△(E5)
△(E6p22)△(E7)
△(E7)▼(E8q5)
△(E6p22)
△(E7_E8)
c.874C>A
△(E7)
mgFL
△(E6p22)△(E7)
△(E6p22)
△(E7_E8)
△(E7)▼(E8q5)
c.876G>T
mgFL
△(E7)
△(E7)▼(E8q5)
△(E6p22)△(E7)
△(E6p22)
△(E7_E8)
Supplementary Figure S3. A) Splicing assays of spliceogenic variants - I.

## Slide 2
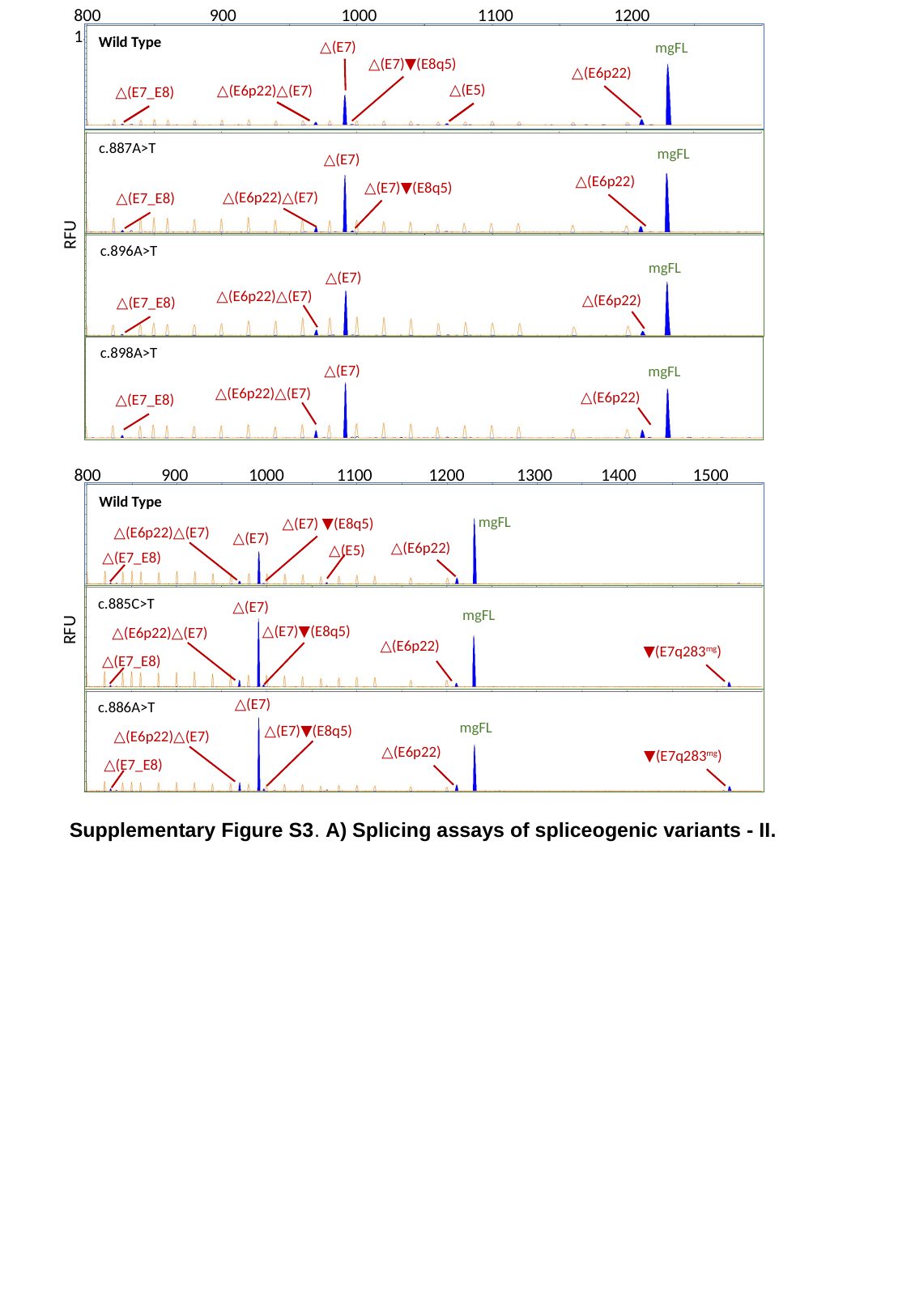

800 900 1000 1100 1200 1300
Wild Type
△(E7)
mgFL
△(E7)▼(E8q5)
△(E6p22)
△(E5)
△(E6p22)△(E7)
△(E7_E8)
c.887A>T
mgFL
△(E7)
△(E6p22)
△(E7)▼(E8q5)
△(E6p22)△(E7)
△(E7_E8)
RFU
c.896A>T
mgFL
△(E7)
△(E6p22)△(E7)
△(E6p22)
△(E7_E8)
c.898A>T
△(E7)
mgFL
△(E6p22)△(E7)
△(E6p22)
△(E7_E8)
800 900 1000 1100 1200 1300 1400 1500
Wild Type
mgFL
△(E7) ▼(E8q5)
△(E6p22)△(E7)
△(E7)
△(E6p22)
△(E5)
△(E7_E8)
c.885C>T
△(E7)
mgFL
△(E7)▼(E8q5)
△(E6p22)△(E7)
△(E6p22)
▼(E7q283mg)
△(E7_E8)
△(E7)
c.886A>T
mgFL
△(E7)▼(E8q5)
△(E6p22)△(E7)
△(E6p22)
▼(E7q283mg)
△(E7_E8)
RFU
Supplementary Figure S3. A) Splicing assays of spliceogenic variants - II.

## Slide 3
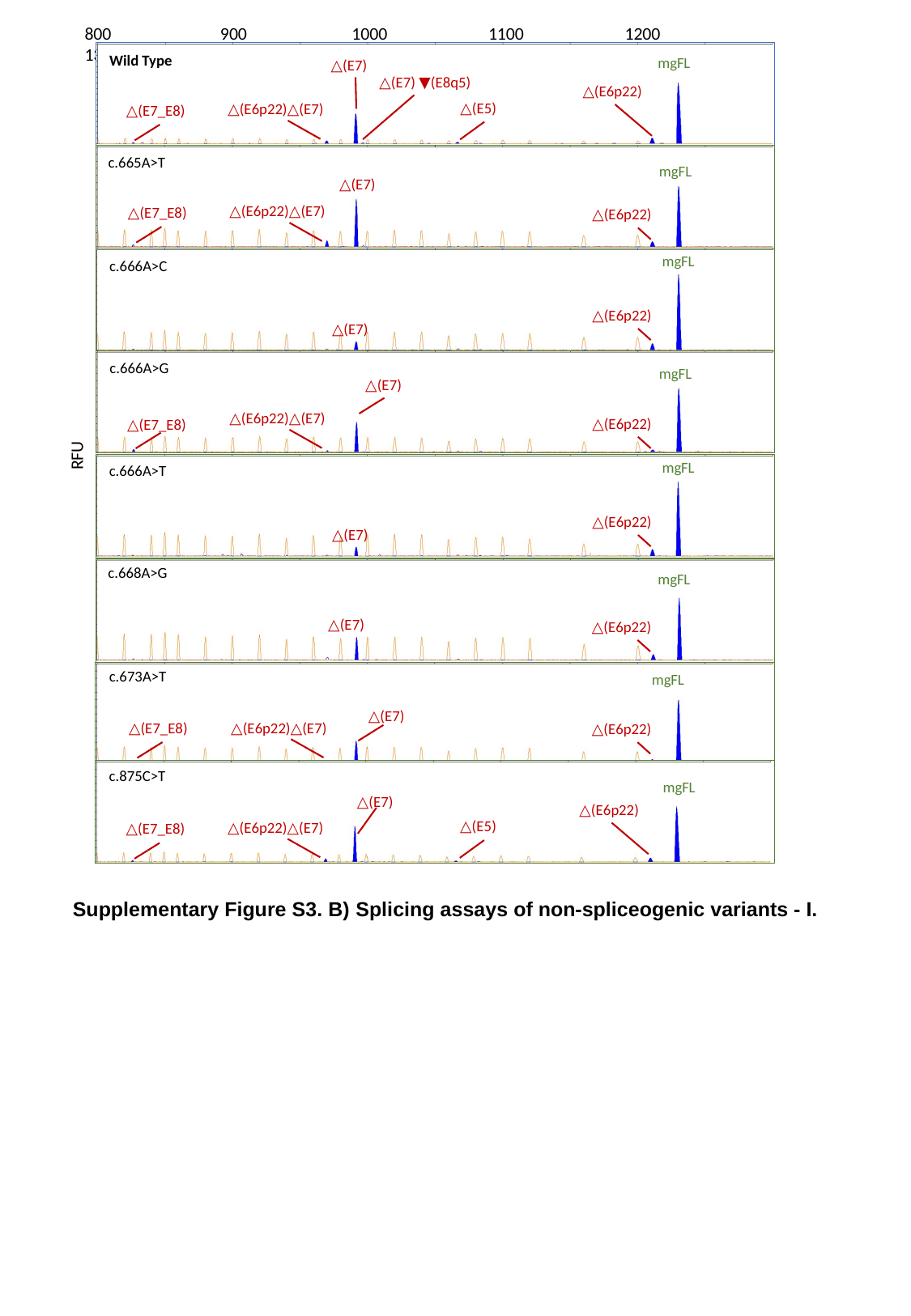

800 900 1000 1100 1200 1300
Wild Type
mgFL
△(E7)
△(E7) ▼(E8q5)
△(E6p22)
△(E5)
△(E6p22)△(E7)
△(E7_E8)
c.665A>T
mgFL
△(E7)
△(E6p22)△(E7)
△(E7_E8)
△(E6p22)
mgFL
c.666A>C
△(E6p22)
△(E7)
c.666A>G
mgFL
△(E7)
△(E6p22)△(E7)
△(E6p22)
△(E7_E8)
mgFL
c.666A>T
△(E6p22)
△(E7)
c.668A>G
mgFL
△(E7)
△(E6p22)
c.673A>T
mgFL
△(E7)
△(E7_E8)
△(E6p22)△(E7)
△(E6p22)
c.875C>T
mgFL
△(E7)
△(E6p22)
△(E5)
△(E6p22)△(E7)
△(E7_E8)
RFU
Supplementary Figure S3. B) Splicing assays of non-spliceogenic variants - I.

## Slide 4
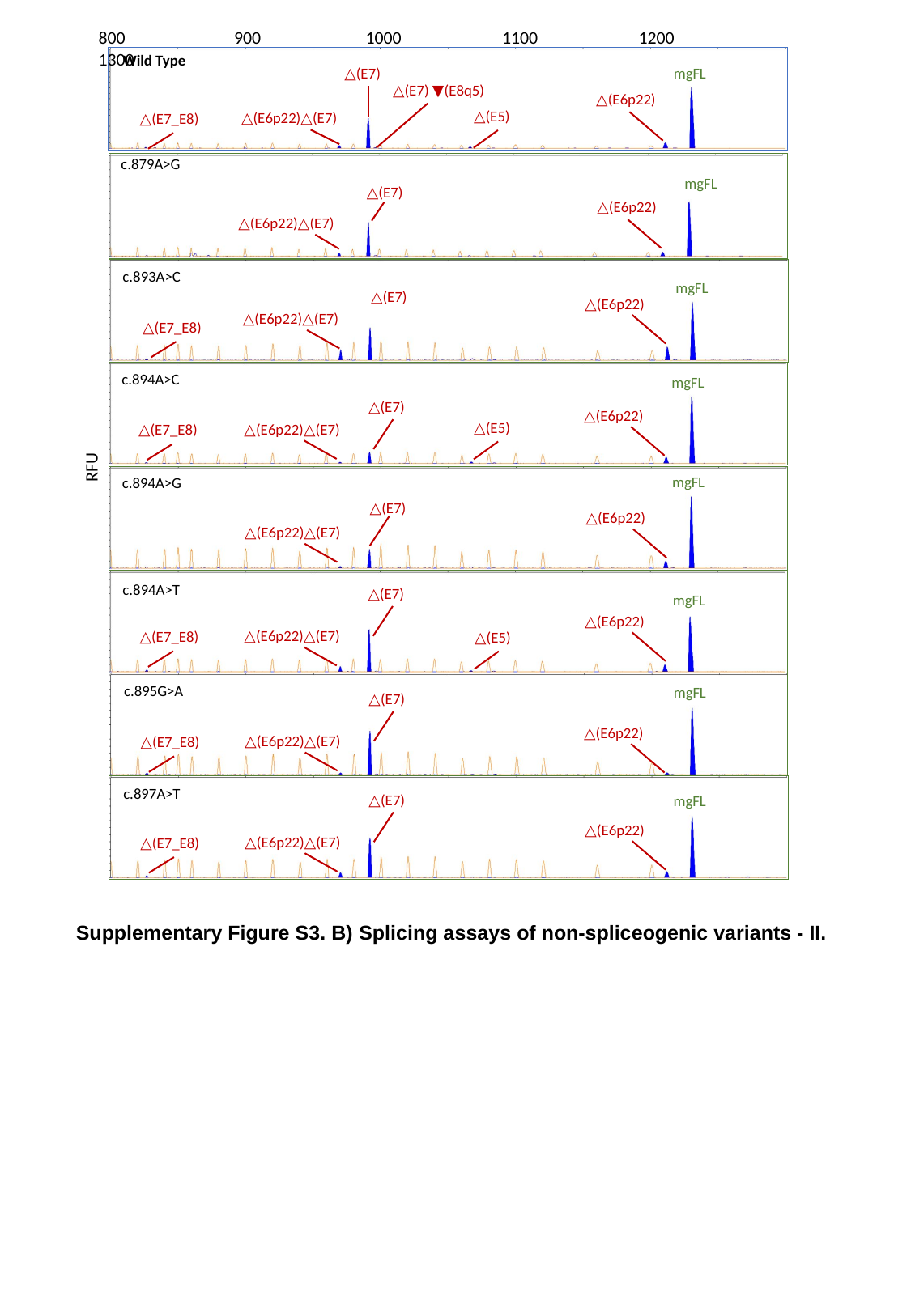

800 900 1000 1100 1200 1300
Wild Type
△(E7)
mgFL
△(E7) ▼(E8q5)
△(E6p22)
△(E5)
△(E6p22)△(E7)
△(E7_E8)
c.879A>G
mgFL
△(E7)
△(E6p22)
△(E6p22)△(E7)
c.893A>C
mgFL
△(E7)
△(E6p22)
△(E6p22)△(E7)
△(E7_E8)
c.894A>C
mgFL
△(E7)
△(E5)
△(E6p22)△(E7)
△(E7_E8)
mgFL
c.894A>G
△(E7)
△(E6p22)△(E7)
c.894A>T
△(E7)
mgFL
△(E6p22)△(E7)
△(E7_E8)
△(E5)
c.895G>A
mgFL
△(E7)
△(E6p22)△(E7)
△(E7_E8)
c.897A>T
△(E7)
mgFL
△(E6p22)△(E7)
△(E7_E8)
△(E6p22)
△(E6p22)
△(E6p22)
△(E6p22)
△(E6p22)
RFU
Supplementary Figure S3. B) Splicing assays of non-spliceogenic variants - II.

## Slide 5
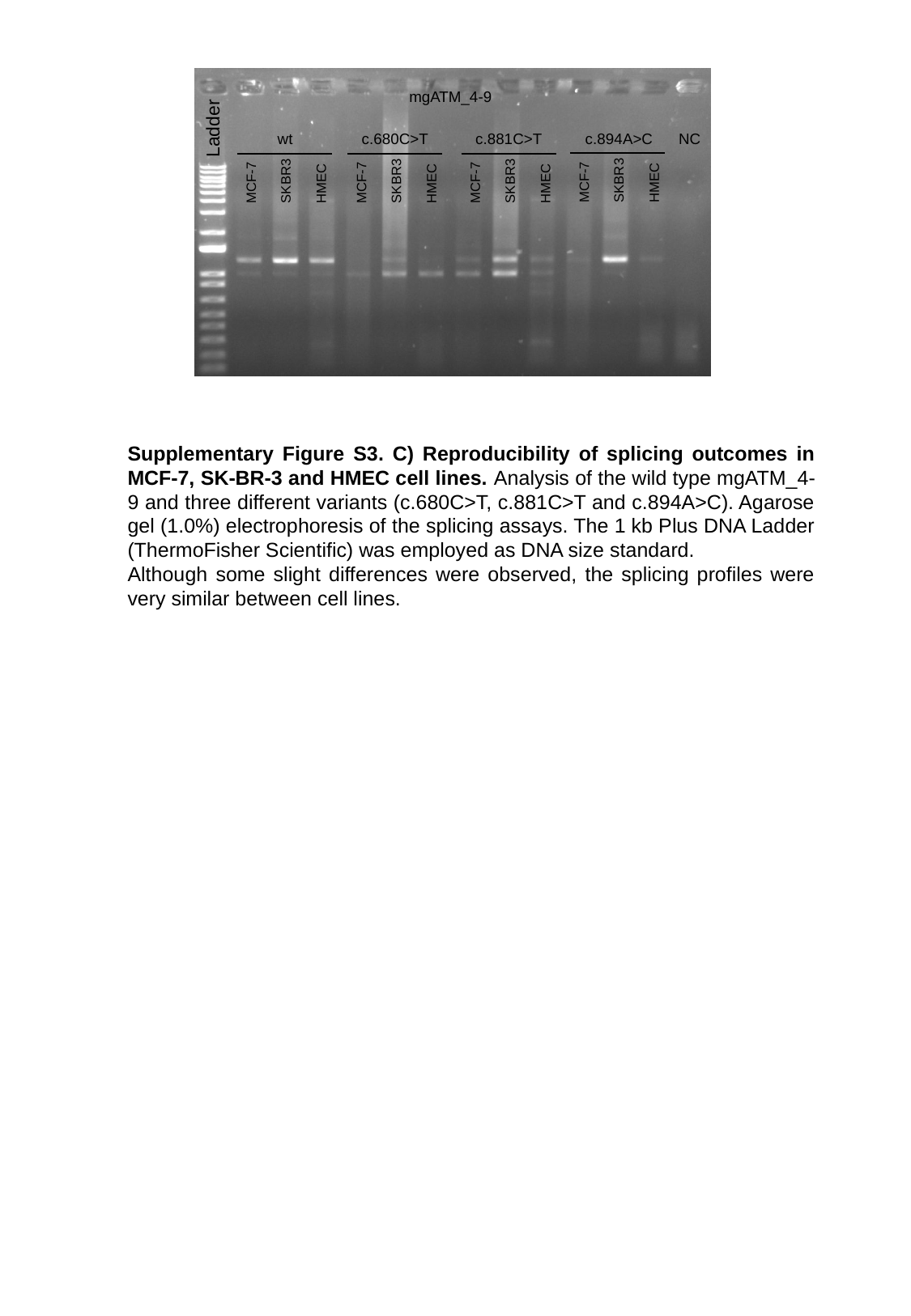

mgATM_4-9
Ladder
wt
c.680C>T
c.881C>T
c.894A>C
NC
SKBR3
HMEC
SKBR3
HMEC
SKBR3
HMEC
SKBR3
HMEC
MCF-7
MCF-7
MCF-7
MCF-7
Supplementary Figure S3. C) Reproducibility of splicing outcomes in MCF-7, SK-BR-3 and HMEC cell lines. Analysis of the wild type mgATM_4-9 and three different variants (c.680C>T, c.881C>T and c.894A>C). Agarose gel (1.0%) electrophoresis of the splicing assays. The 1 kb Plus DNA Ladder (ThermoFisher Scientific) was employed as DNA size standard.
Although some slight differences were observed, the splicing profiles were very similar between cell lines.
