# Supplementary material for: Unproductive alternative splicing of ATM exon 7: mapping of critical regulatory elements and identification of 34 spliceogenic variants
Source: J Mol Med (Berl). 2025 Sep 20;103(11-12):1447–60. doi: 10.1007/s00109-025-02595-0 (PMC12675606; doi:10.1007/s00109-025-02595-0)
Supplement: Supplementary file 4 — Supplementary file4 (DOCX 22 KB) [file 109_2025_2595_MOESM4_ESM.docx]

| **Microdeletions** | **Primers (5’🡪3’)** |
| --- | --- |
| c.665_681del | TGAGCTTGTTTGTTTCTTCACAGACGGTCTAAATCATATCTTAGCAGCTC  GAGCTGCTAAGATATGATTTAGACCGTCTGTGAAGAAACAAACAAGCTCA |
| c.867_898del | AATTATTTCAACTGCAAATTTATATAAGGTATAAAGGAAATGTTTACTGT  ACAGTAAACATTTCCTTTATACCTTATATAAATTTGCAGTTGAAATAATT |

**Supplementary Table S1.** Mutagenesis primers for microdeletions and variants.

| **Variants^1^** | **Protein** | **Primers (5’🡪3’)** |
| --- | --- | --- |
| c.663-2A>G | - | GAGCTTGTTTGTTTCTTCAC**G**GACAAGAAAAGAGCTCTTCA  TGAAGAGCTCTTTTCTTGTC**C**GTGAAGAAACAAACAAGCTC |
| c.665A>T | p.(Gln222Leu) | TTGTTTGTTTCTTCACAGAC**T**AGAAAAGAGCTCTTCAGGTC  GACCTGAAGAGCTCTTTTCT**A**GTCTGTGAAGAAACAAACAA |
| c.666A>B | p.(Gln222His) (A>C/T)  p.(Gln222=) (A>G) | TGTTTGTTTCTTCACAGACA**B**GAAAAGAGCTCTTCAGGTCT  AGACCTGAAGAGCTCTTTTC**V**TGTCTGTGAAGAAACAAACA |
| c.667G>H | p.(Glu223Lys) (G>A)  p.(Glu223Gln) (G>C)  p.(Glu223Ter) (G>T) | GTTTGTTTCTTCACAGACAA**H**AAAAGAGCTCTTCAGGTCTA  TAGACCTGAAGAGCTCTTTT**D**TTGTCTGTGAAGAAACAAAC |
| c.668A>K | p.(Glu223Gly) (A>G)  p.(Glu223Val) (A>T) | TTTGTTTCTTCACAGACAAG**K**AAAGAGCTCTTCAGGTCTAA  TTAGACCTGAAGAGCTCTTT**M**CTTGTCTGTGAAGAAACAAA |
| c.669A>T | p.(Glu223Asp) | TTGTTTCTTCACAGACAAGA**T**AAGAGCTCTTCAGGTCTAAA  TTTAGACCTGAAGAGCTCTT**A**TCTTGTCTGTGAAGAAACAA |
| c.670A>T | p.(Lys224Ter) | TGTTTCTTCACAGACAAGAA**T**AGAGCTCTTCAGGTCTAAAT  ATTTAGACCTGAAGAGCTCT**A**TTCTTGTCTGTGAAGAAACA |
| c.671A>T | p.(Lys224Met) | GTTTCTTCACAGACAAGAAA**T**GAGCTCTTCAGGTCTAAATC  GATTTAGACCTGAAGAGCTC**A**TTTCTTGTCTGTGAAGAAAC |
| c.672G>T | p.(Lys224Asn) | TTTCTTCACAGACAAGAAAA**T**AGCTCTTCAGGTCTAAATCA  TGATTTAGACCTGAAGAGCT**A**TTTTCTTGTCTGTGAAGAAA |
| c.673A>K | p.(Ser225Gly) (A>G)  p.(Ser225Cys) (A>T) | TTCTTCACAGACAAGAAAAG**K**GCTCTTCAGGTCTAAATCAT  ATGATTTAGACCTGAAGAGC**M**CTTTTCTTGTCTGTGAAGAA |
| c.677C>T | p.(Ser226Phe) | TCACAGACAAGAAAAGAGCT**T**TTCAGGTCTAAATCATATCT  AGATATGATTTAGACCTGAA**A**AGCTCTTTTCTTGTCTGTGA |
| c.680C>W | p.(Ser227Ter) (C>A)  p.(Ser227Leu) (C>T) | CAGACAAGAAAAGAGCTCTT**W**AGGTCTAAATCATATCTTAG  CTAAGATATGATTTAGACCT**W**AAGAGCTCTTTTCTTGTCTG |
| c.868C>T | p.(His290Tyr) | TTCAACTGCAAATTTATATC**T**ATCATCCGAAAGGAGCCAAA  TTTGGCTCCTTTCGGATGAT**A**GATATAAATTTGCAGTTGAA |
| c.869A>Y | p.(His290Pro) (A>C)  p.(His290Leu) (A>T) | TCAACTGCAAATTTATATCC**Y**TCATCCGAAAGGAGCCAAAA  TTTTGGCTCCTTTCGGATGA**R**GGATATAAATTTGCAGTTGA |
| c.871C>W | p.(His291Asn) (C>A)  p.(His291Tyr) (C>T) | AACTGCAAATTTATATCCAT**W**ATCCGAAAGGAGCCAAAACC  GGTTTTGGCTCCTTTCGGAT**W**ATGGATATAAATTTGCAGTT |
| c.872A>Y | p.(His291Pro) (A>C)  p.(His291Leu) (A>T) | ACTGCAAATTTATATCCATC**Y**TCCGAAAGGAGCCAAAACCC  GGGTTTTGGCTCCTTTCGGA**R**GATGGATATAAATTTGCAGT |
| c.874C>A | p.(Pro292Thr) | TGCAAATTTATATCCATCAT**A**CGAAAGGAGCCAAAACCCAA  TTGGGTTTTGGCTCCTTTCG**T**ATGATGGATATAAATTTGCA |
| c.875C>T | p.(Pro292Leu) | GCAAATTTATATCCATCATC**T**GAAAGGAGCCAAAACCCAAG  CTTGGGTTTTGGCTCCTTTC**A**GATGATGGATATAAATTTGC |
| c.876G>T | p.(Pro292=) | CAAATTTATATCCATCATCC**T**AAAGGAGCCAAAACCCAAGA  TCTTGGGTTTTGGCTCCTTT**A**GGATGATGGATATAAATTTG |
| c.877A>T | p.(Lys293Ter) | AAATTTATATCCATCATCCG**T**AAGGAGCCAAAACCCAAGAA  TTCTTGGGTTTTGGCTCCTT**A**CGGATGATGGATATAAATTT |
| c.878A>T | p.(Lys293Ile) | AATTTATATCCATCATCCGA**T**AGGAGCCAAAACCCAAGAAA  TTTCTTGGGTTTTGGCTCCT**A**TCGGATGATGGATATAAATT |
| c.879A>G | p.(Lys293=) | ATTTATATCCATCATCCGAA**G**GGAGCCAAAACCCAAGAAAA  TTTTCTTGGGTTTTGGCTCC**C**TTCGGATGATGGATATAAAT |
| c.881G>T | p.(Gly294Val) | TTATATCCATCATCCGAAAG**T**AGCCAAAACCCAAGAAAAAG  CTTTTTCTTGGGTTTTGGCT**A**CTTTCGGATGATGGATATAA |
| c.882A>K | p.(Gly294=) (A>G/T) | TATATCCATCATCCGAAAGG**K**GCCAAAACCCAAGAAAAAGG  CCTTTTTCTTGGGTTTTGGC**M**CCTTTCGGATGATGGATATA |
| c.885C>T | p.(Ala295=) | ATCCATCATCCGAAAGGAGC**T**AAAACCCAAGAAAAAGGTAT  ATACCTTTTTCTTGGGTTTT**A**GCTCCTTTCGGATGATGGAT |
| c.886A>T | p.(Lys296Ter) | TCCATCATCCGAAAGGAGCC**T**AAACCCAAGAAAAAGGTATA  TATACCTTTTTCTTGGGTTT**A**GGCTCCTTTCGGATGATGGA |
| c.887A>T | p.(Lys296Ile) | CCATCATCCGAAAGGAGCCA**T**AACCCAAGAAAAAGGTATAA  TTATACCTTTTTCTTGGGTT**A**TGGCTCCTTTCGGATGATGG |
| c.892C>T | p.(Gln298Ter) | ATCCGAAAGGAGCCAAAACC**T**AAGAAAAAGGTATAAAGGAA  TTCCTTTATACCTTTTTCTT**A**GGTTTTGGCTCCTTTCGGAT |
| c.893A>Y | p.(Gln298Pro) (A>C)  p.(Gln298Leu) (A>T) | TCCGAAAGGAGCCAAAACCC**Y**AGAAAAAGGTATAAAGGAAA  TTTCCTTTATACCTTTTTCT**R**GGGTTTTGGCTCCTTTCGGA |
| c.894A>B | p.(Gln298His) (A>C/T)  p.(Gln298=) (A>G) | CCGAAAGGAGCCAAAACCCA**B**GAAAAAGGTATAAAGGAAAT  ATTTCCTTTATACCTTTTTC**V**TGGGTTTTGGCTCCTTTCGG |
| c.895G>W | p.(Glu299Lys) (G>A)  p.(Glu299Ter) (G>T) | CGAAAGGAGCCAAAACCCAA**W**AAAAAGGTATAAAGGAAATG  CATTTCCTTTATACCTTTTT**W**TTGGGTTTTGGCTCCTTTCG |
| c.896A>T | p.(Glu299Val) | GAAAGGAGCCAAAACCCAAG**T**AAAAGGTATAAAGGAAATGT  ACATTTCCTTTATACCTTTT**A**CTTGGGTTTTGGCTCCTTTC |
| c.897A>T | p.(Glu299Asp) | AAAGGAGCCAAAACCCAAGA**T**AAAGGTATAAAGGAAATGTT  AACATTTCCTTTATACCTTT**A**TCTTGGGTTTTGGCTCCTTT |
| c.898A>T | p.(Lys300Ter) | AAGGAGCCAAAACCCAAGAA**T**AAGGTATAAAGGAAATGTTT  AAACATTTCCTTTATACCTT**A**TTCTTGGGTTTTGGCTCCTT |
| c.901+2T>C | - | GAGCCAAAACCCAAGAAAAAGG**C**ATAAAGGAAATGTTT  AAACATTTCCTTTAT**G**CCTTTTTCTTGGGTTTTGGCTC |

^1^ Ambiguity codes: B=C/G/T; H=A/C/T; K=G/T; W=A/T; Y=C/T
