# Supplementary material for: Unproductive alternative splicing of ATM exon 7: mapping of critical regulatory elements and identification of 34 spliceogenic variants
Source: J Mol Med (Berl). 2025 Sep 20;103(11-12):1447–60. doi: 10.1007/s00109-025-02595-0 (PMC12675606; doi:10.1007/s00109-025-02595-0)
Supplement: Supplementary file 5 — Supplementary file5 (DOCX 40 KB) [file 109_2025_2595_MOESM5_ESM.docx]

**Supplementary Table S2.** Splicing outcomes of microdeletions and SRE-variants of *ATM* exon 7 (full description).

| **Microdel.** | **HEXplorer**  **Score** | **mgFL-transcript** | **Transcripts^1^** |
| --- | --- | --- | --- |
| **mgATM_4-9** |  | 57.6%±1.5% | **△(E7)-transcripts:** △(E7) [30.6%±1.1%] / [△(E6p22)△(E7)] [2.8%±0.1%] / [△(E7)▼(E8q5)] [1%±0.2%] / △(E7_E8) [1.2%±0.1%]  **Other transcripts:** △(E6p22) [5.5%±0.2%] / △(E5) [1.3%±0.1%] |
| c.665_681del |  | 34.6%±1.3% | **△(E7)-transcripts:** △(E7) [45.3%±1.3%] / [△(E6p22)△(E7)] [4.4%±0.2%] / [△(E7)▼(E8q9)] [3.2%±0.6%] /  [△(E7)▼(E8q5)] [1%±0.3%] / △(E7_E8) [2.8%±0.1%]  **Other transcripts:** △(E6p22) [5.7%±0.3%] / △(E5) [3%±0.1%] |
| c.867_898del |  | 2.8%±0.1% | **△(E7)-transcripts:** △(E7 ) [84.2%±0.9%] / [△(E6p22)△(E7)] [10%±0.7%] / [△(E7)▼(E8q5)] [1.8%±0.5%] / △(E7_E8) [1.2%±0.1%] |
| **Variants^2^** |  |  |  |
| ***c.663-2A>G** |  | - | **△(E7)-transcripts:** △(E7) [79.2%±2.4%] / [△(E6p22)△(E7)] [9.5%±0.1%] / ▼(E7p79) [8.6%±2.7%] / [△(E7)▼(E8q5)] [2.7%±0.2%] |
| c.665A>T | -117.5 | 48.8%±0.9% | **△(E7)-transcripts:** △(E7) [40.4%±0.7%] / [△(E6p22)△(E7)] [5.3%±0.2%] / △(E7_E8) [1.5%±0.4%]  **Other transcripts:** △(E6p22) [4%±0.5%] |
| c.666A>C | -55.6 | 81.5%±1.3% | **△(E7)-transcripts:** △(E7) [11.5%±1.3%]  **Other transcripts:** △(E6p22) [7%±0.2%] |
| c.666A>G | -65.6 | 63%±0.8% | **△(E7)-transcripts:** △(E7) [30.9%±0.8%] / [△(E6p22)△(E7)] [1.2%±0.3%] / △(E7_E8) [2.8%±0.1%]  **Other transcripts:** △(E6p22) [2.1%±0.1%] |
| c.666A>T | -64.8 | 84.8%±2% | **△(E7)-transcripts:** △(E7) [8%±2.5%]  **Other transcripts:** △(E6p22) [7.2%±1.2%] |
| *c.667G>A | -113 | 32.4%±2% | **△(E7)-transcripts:** △(E7) [60.6%±2.2%] / [△(E6p22)△(E7)] [3.3%±0.2%] / △(E7_E8) [2.4%±0.3%]  **Other transcripts:** △(E6p22) [1.3%±0.4%] |
| ***c.667G>C** | -40.6 | 23.1%±0.8% | **△(E7)-transcripts:** △(E7) [61.5%±0.7%] / [△(E6p22)△(E7)] [9.2%±0.1%] / △(E7_E8) [2.2%±0.1%] / [△(E7)▼(E8q5)] [1.5%±0.3%]  **Other transcripts:** △(E6p22) [2.5%±0.1%] |
| ***c.667G>T** | -136.6 | 25.4%±0.4% | **△(E7)-transcripts:** △(E7) [60%±0.6%] / [△(E6p22)△(E7)] [7.7%±0.6%] / △(E7_E8) [3.4%±0.3%]  **Other transcripts:** △(E6p22) [3.5%±0.1%] |
| c.668A>G | -42 | 66.2%±2.5% | **△(E7)-transcripts:** △(E7) [28%±2.7%]  **Other transcripts:** △(E6p22) [5.8%±0.5%] |
| ***c.668A>T** | -112 | 12.9%±0.2% | **△(E7)-transcripts:** △(E7) [73.7%±0.3%] / [△(E6p22)△(E7)] [10.5%±0.2%] / △(E7_E8) [1.9%±0.1%] / [△(E7)▼(E8q5)] [1%±0.3%] |
| ***c.669A>T** | -84.4 | 17.6%±1% | **△(E7)-transcripts:** △(E7) [69%±0.9%] / [△(E6p22)△(E7)] [9.5%±0.3%] / △(E7_E8) [2.5%]  **Other transcripts:** △(E6p22) [1.4%±0.1%] |
| ***c.670A>T** | -99.5 | 24.1%±0.1% | **△(E7)-transcripts:** △(E7) [60.9%±0.2%] / [△(E6p22)△(E7)] [8.4%±0.2%] / △(E7_E8) [3.2%±0.1%]  **Other transcripts:** △(E6p22) [3.4%±0.1%] |
| *c.671A>T | -42.4 | 45.8%±2.1% | **△(E7)-transcripts:** △(E7) [43.7%±2.1%] / [△(E6p22)△(E7)] [5.6%±0.3%] / △(E7_E8) [1.6%±0.2%]  **Other transcripts:** △(E6p22) [3.3%±0.1%] |
| ***c.672G>T** | -103.4 | 25.8%±0.2% | **△(E7)-transcripts:** △(E7) [60.1%±0.2%] / [△(E6p22)△(E7)] [8.3%±0.2%] / △(E7_E8) [2.6%] / △(E7)▼(E8q5) [1.2%±0.3%]  **Other transcripts:** △(E6p22) [2%±0.1%] |
| *c.673A>G | -72.8 | 37%±1.9% | **△(E7)-transcripts:** △(E7) [54.8%±1.9%] / △(E6p22)△(E7) [5.5%±1.7%]  **Other transcripts:** △(E6p22) [2.7%±0.1%] |
| c.673A>T | -45.5 | 65.5%±0.4% | **△(E7)-transcripts:** △(E7) [24.8%±0.3%] / [△(E6p22)△(E7)] [2.4%±0.6%] / △(E7_E8) [2.1%±0.8%]  **Other transcripts:** △(E6p22) [5.2%±0.4%] |
| ***c.677C>T** | -93.4 | 19.1%±0.7% | **△(E7)-transcripts:** △(E7) [67.5%±0.7%] / [△(E6p22)△(E7)] [8.4%±0.2%] / △(E7_E8) [2.1%±0.4%] / [△(E7)▼(E8q5)] [1.6%±0.1%]  **Other transcripts:** △(E6p22) [1.3%±0.4%] |
| ***c.680C>A** | -53.3 | 5.5%±0.4% | **△(E7)-transcripts:** △(E7) [14%±0.7%] / △(E7_E8) [70.2%±0.7%] / [△(E6p22)△(E7_E8)] [6.8%±0.4%]  **Other transcripts:** △(E5) [3.5%±0.1%] |
| ***c.680C>T** | -90.9 | 3.9%±0.2% | **△(E7)-transcripts:** △(E7) [80.4%±0.7%] / [△(E6p22)△(E7)] [12.3%±0.3%] / [△(E7)▼(E8q5)] [1.8%±0.1%] /  △(E7_E8) [1.6%±0.1%] |
| *c.868C>T | -48.2 | 43.1%±0.5% | **△(E7)-transcripts:** △(E7) [50.4%±1.3%] / [△(E6p22)△(E7)] [5.1%±1.3%] / △(E7_E8) [1.4%±0.1%] |
| *c.869A>C | -55.8 | 33.8%±0.2% | **△(E7)-transcripts:** △(E7) [56.4±0.3%] / [△(E6p22)△(E7)] [5.9%±0.2%] / △(E7_E8) [1.4%±0.1%]  **Other transcripts:** △(E6p22) [2.5%±0.1%] |
| *c.869A>T | -42.2 | 44.5%±1% | **△(E7)-transcripts:** △(E7) [46.6%±1%] / [△(E6p22)△(E7)] [4.5%±0.1%] / △(E7_E8) [1.4%±0.1%]  **Other transcripts:** △(E6p22) [3%±0.1%] |
| ***c.871C>A** | -95.8 | 25.8%±0.8% | **△(E7)-transcripts:** △(E7) [62%±0.3%] / [△(E6p22)△(E7)] [7.3%±0.1%] / [△(E7)▼(E8q5)] [2.1%±0.2%] / △(E7_E8) [1.3%±0.5]  **Other transcripts:** △(E6p22) [1.5%] |
| ***c.871C>T** | -72.6 | 29.1%±1.3% | **△(E7)-transcripts:** △(E7) [58.6%±1.2%] / [△(E6p22)△(E7)] [6.8%±0.4%] / [△(E7)▼(E8q5)] [1.8%±0.1%] / △(E7_E8) [1.7%±0.2%]  **Other transcripts:** △(E6p22) [2%±0.5%] |
| *c.872A>C | -45.1 | 31.4%±1.7% | **△(E7)-transcripts:** △(E7) [63.4%±2.6%] / [△(E6p22)△(E7)] [2.7%±0.1%] / [△(E7)▼(E8q5)] [1.2%±0.5%] / △(E7_E8) [1.3%±0.3%] |
| *c.872A>T | -44.7 | 43%±0.9% | **△(E7)-transcripts:** △(E7) [45.1%±0.8%] / [△(E6p22)△(E7)] [4.5%±0.1%] / [△(E7)▼(E8q5)] [1.2%±0.1%] / △(E7_E8) [1.6%]  **Other transcripts:** △(E6p22) [3.5%±0.2%] / △(E5) [1.1%] |
| *c.874C>A | -50.4 | 36.9%±1.3% | **△(E7)-transcripts:** △(E7) [50.4%±1.2%] / [△(E6p22)△(E7)] [5.4%±0.1%] / [△(E7)▼(E8q5)] [1.8%±0.3%] / △(E7_E8) [1.7%±0.2%]  **Other transcripts:** △(E6p22) [3.8%±0.2%] |
| c.875C>T | -43.1 | 56.4%±0.8% | **△(E7)-transcripts:** △(E7) [34.5%±0.5%] / [△(E6p22)△(E7)] [3%±0.1%] / △(E7_E8) [1.2%±0.4%]  **Others:** △(E6p22) [3.7%±0.4%] / △(E5) [1.2%±0.1%] |
| *c.876G>T | -62.9 | 47.2%±0.9% | **△(E7)-transcripts:** △(E7) [43.5%±1.4%] / [△(E6p22)△(E7)] [4.1%±0.1%] / △(E7_E8) [1.3%±0.1%] / [△(E7)▼(E8q5)] [1.1%±0.3%]  **Others:** △(E6p22) [2.8%±0.7%] |
| ***c.877A>T** | -45.7 | 27.8%±0.5% | **△(E7)-transcripts:** △(E7) [55.4%±1.8%] / [△(E6p22)△(E7)] [5.8%±0.1%] / [△(E7)▼(E8q5)] [1.7%±0.5%] / △(E7_E8) [1.7%±0.1%]  **Others:** △(E6p22) [3.2%±0.2%] / ▼(E7q283^mg^) [4.4%±0.8%] |
| ***c.878A>T** | -66.8 | 29.2%±0.1% | **△(E7)-transcripts:** △(E7) [55.9%±0.2%] / [△(E6p22)△(E7)] [5.8%±0.1%] / △(E7_E8) [1.4%] [△(E7)▼(E8q5)] [1.3%±0.1%]  **Others:** ▼(E7q283^mg^) [4.6%±0.1%] / △(E6p22) [1.8%±0.2%] |
| c.879A>G^3^ | -69.2 | 60.4%±2.9% | **△(E7)-transcripts:** △(E7) [30.6%±1.7%] / [△(E6p22)△(E7)] [2.6%±0.5%]  **Others:** △(E6p22) [4%±0.3%] / |
| ***c.881G>T** | -79 | 15.9%±1.5% | **△(E7)-transcripts:** △(E7) [69.2%±2.4%] / [△(E6p22)△(E7)] [7.9%±0.1%] / [△(E7)▼(E8q5)] [1.8%±0.3%] / △(E7_E8) [1.5%±0.1%]  **Others:** ▼(E7q283^mg^) [3.7%±0.7%] |
| ***c.882A>G** | -99.8 | 24.5%±0.6% | **△(E7)-transcripts:** △(E7) [59.9%±0.6%] / [△(E6p22)△(E7)] [6.6%0.3%] / [△(E7)▼(E8q5)] [1.9%±0.4%] / △(E7_E8) [1.3%]  **Others:** △(E6p22) [1.6%±0.1%] / ▼(E7q283^mg^) [4.2%±0.3%] |
| ***c.882A>T** | -46.7 | 26.7%±0.7% | **△(E7)-transcripts:** △(E7) [58.2%±0.5%] / [△(E6p22)△(E7)] [6.4%0.1%] / [△(E7)▼(E8q5)] [1.4%±0.3%] / △(E7_E8) [1.4%±0.1%]  **Others:** △(E6p22) [1.8%±0.3%] / ▼(E7q283^mg^) [4.1%±0.2%] |
| *c.885C>T | -40.6 | 36.2%±1.5% | **△(E7)-transcripts:** △(E7) [49.8%±1.6%] / [△(E6p22)△(E7)] [5%±0.1%] / [△(E7)▼(E8q5)] [1.5%±0.2%] / △(E7_E8) [1.3%±0.1%]  **Others:** △(E6p22) [2.8%±0.1%] / ▼(E7q283^mg^) [3.4%±0.3%] |
| *c.886A>T | -54.4 | 30.7%±1.6% | **△(E7)-transcripts:** △(E7) [52.1%±1.1%] / [△(E6p22)△(E7)] [6%±0.3%] / △(E7_E8) [2%±0.2%] / [△(E7)▼(E8q5)] [1.6%±0.2%]  **Others:** △(E6p22) [4.5%±0.2%] / ▼(E7q283^mg^) [3.1%±0.3%] |
| *c.887A>T | -46.1 | 45.1%±0.9% | **△(E7)-transcripts:** △(E7) [43.3%±0.8%] / [△(E6p22)△(E7)] [4%] / [△(E6_E7)▼(E8q5)] [1.6%±0.1] / △(E7_E8) [1.5%±0.1%]  **Others:** △(E6p22) [4.5%±0.2%] |
| ***c.892C>T** | -71.5 | 21.5%±0.7% | **△(E7)-transcripts:** △(E7) [69.7%±0.3%] / [△(E6p22)△(E7)] [3.8%±0.1%] / △(E7_E8) [2.5%±0.1%] / [△(E7)▼(E8q5)] [1.2%±0.6%]  **Others:** △(E6p22) [1.3%±0.1%] |
| c.893A>C | -43 | 52.9%±1.6% | **△(E7)-transcripts:** △(E7) [26.1%±1.9%] / [△(E6p22)△(E7)] [9%±0.1%]  **Others:** △(E6p22) [12%±0.4%] |
| ***c.893A>T** | -104.7 | 27.1%±0.6% | **△(E7)-transcripts:** △(E7) [61.1%±0.5%] / [△(E6p22)△(E7)] [7.9%±0.2%] / △(E7_E8) [1.4%±0.4%]  **Others:** △(E6p22) [2.5%±0.1%] |
| c.894A>C | -43.3 | 75.7%±0.2% | **△(E7)-transcripts:** △(E7) [13.4%±0.2%] / [△(E6p22)△(E7)] [1.5%±0.1%] / △(E7_E8) [1.1%±0.4%]  **Others:** △(E6p22) [6.7%±0.3%] / △(E5) [1.6%±0.1%] |
| c.894A>G | -46.8 | 71.5%±0.6% | **△(E7)-transcripts:** △(E7) [18.6%±0.9%] / [△(E6p22)△(E7)] [2.4%±0.5%]  **Others:** △(E6p22) [7.5%±0.4%] |
| c.894A>T | -59.2 | 50.7%±3.1% | **△(E7)-transcripts:** △(E7) [35.8%±2.7%] / [△(E6p22)△(E7)] [4.6%±0.3%] / △(E7_E8) [1.7%±0.1%]  **Others:** △(E6p22) [6.1%±0.2%] / △(E5) [1.1%±0.1%] |
| c.895G>A | -126.2 | 58.3%±2.8% | **△(E7)-transcripts:** △(E7) [37.4%±2.4%] / [△(E6p22)△(E7)] [1.5%±0.1%] / △(E7_E8) [1.2%±0.4%]  **Others:** △(E6p22) [1.6%±0.1%] |
| ***c.895G>T** | -138 | 25.4%±1.1% | **△(E7)-transcripts:** △(E7) [45.3%±0.9%] / [△(E6p22)△(E7)] [16.7%±0.4%] / △(E7_E8) [1.4%±0.1%]  **Others:** △(E6p22) [10.2%±0.3%] / △(E5) [1%] |
| *c.896A>T | -116 | 44.9±1.8% | **△(E7)-transcripts:** △(E7) [43.9%±0.8%] / [△(E6p22)△(E7)] [5.6%±0.3%] / △(E7_E8) [1.4%±0.2%]  **Others:** △(E6p22) [4.2%±0.4%] |
| c.897A>T | -67.5 | 50.5%±0.6% | **△(E7)-transcripts:** △(E7) [37.9%±0.7%] / [△(E6p22)△(E7)] [4.7%±0.1%] / △(E7_E8) [1.6%±0.5%]  **Others:** △(E6p22) [5.3%±0.1%] |
| *c.898A>T | -77.5 | 37.7%±2.1% | **△(E7)-transcripts:** △(E7) [47.5%±2.5%] / [△(E6p22)△(E7)] [6.4%±0.2%] / △(E7_E8) [2.2%±0.2%]  **Others:** △(E6p22) [6.2%±0.4%] |
| ***c.901+2T>C^4^** |  | - | **△(E7)-transcripts:** △(E7) [100.0%] |

^1^ PTC-NMD transcripts: △(E7): p.(Gln222Cysfs*3); △(E6p22): p.(Glu166Glyfs*4); △(E7_E8): p.(Gln222Phefs*34); △(E6p22)△(E7): p.(Glu166Glyfs*4); △(E6p22)△(E7_E8): p.(Glu166Glyfs*4); △(E7)▼(E8q5): p.(Gln222Cysfs*3); △(E7)▼(E8q9): p.(Gln222Cysfs*3); △(E6_E7)▼(E8q5): p.(Glu166_Lys300del, Phe357Glnfs*35); ▼(E7q283^mg^): p.(Ala302delinsIleTer). In-frame transcripts: △(E5): p.(Arg111_Glu166delinsLys).

^2^ Spliceogenic variants (mgFL<47.6%) are shown with an asterisk. Variants with <30% mgFL-transcript are shown in bold.

^3^ One uncharacterized transcript was found: 861 nt [2.4%] (c.879A>G)

^4^ Bueno-Martínez at al (2022).
