# Supplementary material for: Unproductive alternative splicing of ATM exon 7: mapping of critical regulatory elements and identification of 34 spliceogenic variants
Source: J Mol Med (Berl). 2025 Sep 20;103(11-12):1447–60. doi: 10.1007/s00109-025-02595-0 (PMC12675606; doi:10.1007/s00109-025-02595-0)
Supplement: Supplementary file 6 — Supplementary file6 (DOCX 48.7 KB) [file 109_2025_2595_MOESM6_ESM.docx]

**Supplementary Table S3.** HEXplorer and SpliceAI predictions and ClinVar classification of all possible single-nucleotide substitutions in *ATM* exon 7 regions c.665–681 and c.867–898.

| **Variant^1^** | **HEXplorer^2^ score** | **ClinVar** | **SpliceAI^4^** | | | |
| --- | --- | --- | --- | --- | --- | --- |
|  |  |  | **AG** | **AL** | **DG** | **DL** |
| c.663-2A>G | 20 | LP | 0.76 (-77) | 0.94 (2) | 0.01 (21) | 0.04 (2350) |
| c.665A>C | -27.1 | VUS | 0.14 (3) | 0.01 (-81) | - | - |
| c.665A>G | -20.1 | VUS | 0.08 (-81) | 0.01(-2) | - | - |
| **c.665A>T** | -117.5 | - | 0.1 (3) | 0.04 (-2) | 0.1 (3) | 0.02 (2346) |
| **c.666A>C** | -55.6 | - | 0.02 (-3) | 0.07 (-82) | - | - |
| **c.666A>G** | -65.6 | LB | 0.42 (1) | 0.03 (-82) | - | 0.01 (2345) |
| **c.666A>T** | -64.8 | - | 0.01 (-3) | 0.06 (-82) | - | - |
| **c.667G>A** | -113 | - | 0.04 (57) | 0.05 (-4) | - | 0.01 (2344) |
| **c.667G>C** | -40.6 | - | 0.02 (-83) | 0.02 (-4) | - | 0.01 (2344) |
| **c.667G>T** | -136.6 | P | 0.08 (57) | 0.08 (-4) | - | 0.02 (2344) |
| c.668A>C | -33.4 | VUS | 0.02 (-84) | 0.01 (-5) | - | 0.01 (2343) |
| **c.668A>G** | -42.1 | VUS | 0.03 (-84) | 0.03 (-5) | - | 0.01 (2343) |
| **c.668A>T** | -112.1 | - | 0.12 (-84) | 0.07 (-5) | - | 0.01 (2343) |
| c.669A>C | -7.5 | - | 0.01 (-6) | 0.02 (-85) | - | - |
| c.669A>G | 25.1 | - | 0.02 (-6) | 0.03 (-85) | 0.01 (2342) | - |
| **c.669A>T** | -84.4 | - | 0.06 (-85) | 0.05 (-6) | - | 0.01 (2342) |
| c.670A>C | -12.4 | - |  | 0.02 (-86) | - | - |
| c.670A>G | 31.7 | VUS/LB^3^ | 0.03 (-7) | 0.03 (-86) | 0.01 (2341) | - |
| **c.670A>T** | -99.5 | - | 0.07 (-86) | 0.13 (-7) | - | 0.03 (2341) |
| c.671A>C | 4 | - | 0.02 (-8) | 0.01 (-87) | - | - |
| c.671A>G | 11.4 | - | 0.01 (-3) | 0.01 (53) | - | - |
| **c.671A>T** | -42.4 | - | 0.02 (53) | 0.01 (-8) | - | - |
| c.672G>A | -38.6 | - | 0.04 (-88) | 0.01 (-9) | - | - |
| c.672G>C | -9.3 | VUS | 0.01 (-88) | - | - | - |
| **c.672G>T** | -103.4 | VUS/LB^3^ | 0.09 (-88) | 0.07 (-9) | - | 0.02 (2339) |
| c.673A>C | -10.9 | VUS | 0.01 (-10) | 0.02 (-89) | - | - |
| **c.673A>G** | -72.8 | - | 0.04 (51) | 0.05 (-10) | - | 0.01 (2338) |
| **c.673A>T** | -45.5 | - | 0.06 (51) | 0.03 (-10) | - | 0.01 (2338) |
| c.674G>A | 2.5 | - | 0.01 (-11) | - | - | - |
| c.674G>C | -0.3 | - | 0.01 (-11) | 0.01 (50) | - | - |
| c.674G>T | -22.7 | - | 0.01 (50) | - | - | - |
| c.675C>A | 22 | VUS | 0.02 (-91) | - | - | - |
| c.675C>G | -30 | VUS | 0.03 (-91) | 0.01 (-12) | - | 0.01 (2336) |
| c.675C>T | -35.7 | LB | 0.04 (-91) | - | - | - |
| c.676T>A | 0.1 | - | 0.01 (-92) | 0.01 (-13) | - | - |
| c.676T>C | -26.3 | - | - | 0.02 (-92) | - | - |
| c.676T>G | 30.1 | - | 0.01 (-13) | 0.01 (-92) | - | - |
| c.677C>A | -29.8 | - | 0.08 (-93) | 0.09 (-14) | - | 0.02 (2334) |
| c.677C>G | -7 | VUS | 0.03 (47) | 0.04 (-14) | - | 0.01 (2334) |
| **c.677C>T** | -93.4 | VUS | 0.08 (-93) | 0.10 (-14) | - | 0.02 (2334) |
| c.678T>A | 32.8 | - | 0.03 (-94) | 0.01 (-15) | - | - |
| c.678T>C | -13.1 | B/LB | - | 0.02 (-94) | - | - |
| c.678T>G | 12 | LB | - | 0.01 (-94) | - | - |
| c.679T>A | 18.5 | - | - | - | - | - |
| c.679T>C | -1.6 | - | 0.01 (-16) | 0.04 (-95) | 0.01 (2332) | - |
| c.679T>G | 5.8 | - | 0.01 (-16) | 0.02 (-95) | - | - |
| **c.680C>A** | -53.3 | LP | 0.09 (44) | 0.10 (-17) | - | 0.02 (2331) |
| c.680C>G | -39.4 | P/LP | 0.05 (-96) | 0.03 (-17) | - | 0.01 (2331) |
| **c.680C>T** | -90.9 | P/VUS^3^ | 0.12 (44) | 0.2 (-17) | - | 0.03 (2331) |
| c.681A>C | 27.1 | - | 0.03 (-18) | 0.02 (-97) | 0.01 (2330) | - |
| c.681A>G | 13 | B/LB | 0.02 (-18) | 0.01 (43) | 0.01 (2330) | - |
| c.681A>T | -9 | - | 0.02 (-97) | 0.01 (43) | 0.01 (2330) | - |
| c.867C>A | -26.7 | LB | - | - | - | 0.01 (2144) |
| c.867C>G | -17.8 | - | - | - | - | - |
| c.867C>T | -28.3 | LB | - | - | - | - |
| c.868C>A | -21 | VUS | - | - | - | - |
| c.868C>G | 1.2 | - | 0.01 (-205) | - | 0.01 (2143) | - |
| **c.868C>T** | -48.2 | VUS | - | 0.001 (-205) | - | 0.01 (2143) |
| **c.869A>C** | -55.8 | VUS | - | 0.02 (-206) | - | 0.01 (2142) |
| c.869A>G | -4.5 | VUS | - |  | - | - |
| **c.869A>T** | -42.2 | - | 0.01 (12) | 0.01 (-206) | - | - |
| c.870T>A | 11.6 | - | - | - | - | - |
| c.870T>C | 3.4 | - | - | - | - | - |
| c.870T>G | 3.4 | VUS | 0.01 (1) | - | - | - |
| **c.871C>A** | -95.8 | - | - | 0.03 (-208) | 0.01 (-121) | 0.01 (2140) |
| c.871C>G | -15.7 | VUS | - | 0.01 (-208) | - | - |
| **c.871C>T** | -72.6 | VUS | - | 0.03 (-208) | 0.01 (-121) | 0.02 (2140) |
| **c.872A>C** | -45.1 | - | - | 0.01 (-209) | - | - |
| c.872A>G | -12.2 | VUS | 0.01 (-209) | - | 0.01 (2139) | - |
| **c.872A>T** | -44.7 | - | - | 0.01 (-209) | - | - |
| c.873T>A | 5.2 | - | - | - | - | - |
| c.873T>C | -17.7 | LB | - | 0.01 (-210) | - | - |
| c.873T>G | -7.9 | - | 0.01 (1) | 0.01 (-210) | - | - |
| **c.874C>A** | -50.4 | VUS | - | 0.01 (-211) | - | 0.01 (2137) |
| c.874C>G | -34.4 | - | - | - | - | - |
| c.874C>T | -38.8 | VUS | - | - | - | 0.01 (2137) |
| c.875C>A | 8.6 | - | 0.01 (2) | 0.01 (-212) | - | 0.01 (2136) |
| c.875C>G | -3 | VUS | 0.01 (-212) | - | 0.01 (213) | - |
| **c.875C>T** | -43.1 | LP | - | 0.01 (-212) | - | 0.01 (2136) |
| c.876G>A | -6.6 | B/LB | - | 0.02 (-213) | - | 0.01 (2135) |
| c.876G>C | -33.5 | - | - | 0.02 (-213) | - | 0.01 (2135) |
| **c.876G>T** | -62.9 | LB | - | 0.02 (-213) | - | 0.01 (2135) |
| c.877A>C | 22.7 | - | - | - | - | - |
| c.877A>G | 22.2 | VUS | 0.01 (-214) | - | 0.01 (2134) | - |
| **c.877A>T** | -45.6 | P/LP | - | 0.03 (-214) | 0.01 (-127) | 0.02 (2134) |
| c.878A>C | -12.4 | - | 0.02 (3) | - | - | - |
| c.878A>G | 12.3 | - | - | - | - | - |
| **c.878A>T** | -66.8 | - | 0.01 (3) | 0.03 (-215) | - | 0.01 (2133) |
| c.879A>C | -13.6 | - | - | - | - | - |
| **c.879A>G** | -69.2 | VUS | - | 0.01 (-216) | - | - |
| c.879A>T | -5.8 | - | - | - | - | - |
| c.880G>A | -1.2 | VUS | - | 0.02 (-217) | - | 0.01 (2131) |
| c.880G>C | -1.1 | - | - | 0.01 (-217) | - | 0.01 (2131) |
| c.880G>T | -38.4 | - | - | 0.08 (-217) | 0.01 (-130) | 0.03 (2131) |
| c.881G>A | 49 | - | 0.01 (-218) | - | - | - |
| c.881G>C | 15.6 | - | - | 0.01 (-218) | 0.01 (-131) | 0.01 (2130) |
| **c.881G>T** | -79 | VUS | - | 0.07 (-218) | 0.02 (-131) | 0.03 (2130) |
| c.882A>C | -25.8 | - | - | - | - | - |
| **c.882A>G** | -99.8 | - | - | 0.05 (-219) | 0.01 (-132) | 0.02 (2129) |
| **c.882A>T** | -46.6 | - | - | 0.02 (-219) | 0.01 (-2) | 0.02 (2129) |
| c.883G>A | -10.7 | - | - | - | - | - |
| c.883G>C | -23.6 | - | - | 0.01 (-220) | - | 0.01 (2128) |
| c.883G>T | 21.01 | - | - | - | - | - |
| c.884C>A | 6.8 | VUS | - | - | - | - |
| c.884C>G | -30.1 | - | - | 0.01 (-221) | - | 0.01 (2127) |
| c.884C>T | -35.2 | VUS | - | - | - | - |
| c.885C>A | 4.6 | - | - | 0.01 (1954) | - | 0.01 (2126) |
| c.885C>G | 16.7 | - | 0.01 (-222) | - | - | - |
| **c.885C>T** | -40.6 | LB | - | 0.01 (-222) | - | 0.01 (2126) |
| c.886A>C | -17 | VUS | - | 0.01 (-223) | - | 0.01 (2125) |
| c.886A>G | 4.9 | VUS | 0.01 (-223) | - | 0.01 (2125) |  |
| **c.886A>T** | -54.4 | - | - | 0.02 (-223) | - | 0.02 (2125) |
| c.887A>C | 4.5 | VUS | 0.01 (-224) | - | - | - |
| c.887A>G | 19.7 | VUS | - | - | - | - |
| **c.887A>T** | -46.1 | - | - | 0.01 (-224) | - | 0.01 (2124) |
| c.888A>C | 15.5 | VUS | 0.01 (-225) | - | 0.01 (2123) | - |
| c.888A>G | 32.5 | LB | 0.01 (-225) | - | - | - |
| c.888A>T | -17 | - | - | - | - | - |
| c.889A>C | -16.5 | - | - | - | - | - |
| c.889A>G | 12 | VUS | - | - | - | - |
| c.889A>T | 24.2 | - | - | - | - | - |
| c.890C>A | 18.7 | - | - | - | - | - |
| c.890C>G | 27.5 | - | - | - | - | - |
| c.890C>T | 2.5 | LB | - | - | - | - |
| c.891C>A | 1.9 | - | - | - | - | - |
| c.891C>G | 16.7 | LB | 0.01 (-228) | - | - | - |
| c.891C>T | -2.8 | - | 0.01 (-228) | - | - | - |
| c.892C>A | 9.5 | - | - | - | - | - |
| c.892C>G | 29.8 | - | 0.01 (-229) | - | 0.01 (2119) | - |
| **c.892C>T** | -71.5 | P | 0.01 (-308) | 0.02 (-229) | - | 0.02 (2119) |
| **c.893A>C** | -43 | VUS | - | 0.01 (-230) | - | 0.01 (2118) |
| c.893A>G | -10.7 | - | - | - | - | - |
| **c.893A>T** | -104.7 | - | - | 0.03 (-230) | 0.01 (-143) | 0.02 (2118) |
| **c.894A>C** | -43.3 | VUS | - | - | - | - |
| **c.894A>G** | -46.8 | LB | - | 0.01 (-231) | - | - |
| **c.894A>T** | -59.2 | - | - | 0.01 (-231) | - | 0.01 (2117) |
| **c.895G>A** | -126.2 | VUS | - | 0.03 (-232) | 0.01 (-145) | 0.01 (6) |
| c.895G>C | -38.7 | VUS | - | 0.01 (-232) | - | - |
| **c.895G>T** | -138 | LP | - | 0.05 (-232) | 0.01 (-145) | 0.02 (2116) |
| c.896A>C | -35.2 | - | - | 0.01 (-233) | - | - |
| c.896A>G | -24.7 | VUS | - | 0.01 (-233) | - | 0.01 (2115) |
| **c.896A>T** | -116 | VUS | - | 0.02 (-233) | 0.02 (-2) | - |
| c.897A>C | -17.8 | - | - | 0.01 (-234) | - | - |
| c.897A>G | -15.9 | LB | - | 0.02 (-234) | - | 0.01 (2114) |
| **c.897A>T** | -67.5 | - | - | 0.01 (-234) | - | - |
| c.898A>C | 23.1 | - | - | - | - | - |
| c.898A>G | 105.6 | VUS | - | 0.01 (-235) | 0.01 (-148) | 0.02 (2113) |
| **c.898A>T** | -77.5 | - | - | 0.02 (-235) | - | 0.01 (2113) |
| c.901+2T>C | 44.8 | P/LP | - | 0.81 (-240) | 0.02 (-153) | 0.99 (-2) |

^1^Tested variants in bold.

^2^ △HZEI score <-40 are shaded.

^3^Conflicting classifications.

^4^SpliceAI parameters: genome version: hg38; score type: raw; max distance: 10000. △ score (and pre-mRNA position) are shown for acceptor gain (AG), acceptor loss (AL), donor gain (DG) and donor loss (DL). For clarity, high precision (△ score >0.80), recommended (△ score >0.50) and high recall (△ score >0.2) have been color-coded red, yellow and green, respectively.
