# Supplementary material for: Unproductive alternative splicing of ATM exon 7: mapping of critical regulatory elements and identification of 34 spliceogenic variants
Source: J Mol Med (Berl). 2025 Sep 20;103(11-12):1447–60. doi: 10.1007/s00109-025-02595-0 (PMC12675606; doi:10.1007/s00109-025-02595-0)
Supplement: Supplementary file 7 — Supplementary file7 (DOCX 25 KB) [file 109_2025_2595_MOESM7_ESM.docx]

|  | c.667G>C | c.667G>T | c.668A>T | c.669A>T | c.670A>T | c.672G>T | c.677C>T | c.680C>A | c.680C>T | c.871C>A | c.871C>T | c.877A>T | c.878A>T | c.881G>T | c.882A>G | c.882A>T | c.892C>T | c.893A>T | c.895G>T |
| --- | --- | --- | --- | --- | --- | --- | --- | --- | --- | --- | --- | --- | --- | --- | --- | --- | --- | --- | --- |
| DAZAP1 |  |  |  |  |  | 0.29 |  |  |  |  |  |  | 0.23 | 0.30 | -0.33 |  |  |  |  |
| hnRNPA1 |  |  |  |  |  |  |  |  | 0.23 |  |  |  |  |  |  |  |  |  |  |
| hnRNPA1L2 |  |  |  |  |  |  | 0.45 | 0.63 | 0.43 | 0.36 | 0.22 |  | -0.26 |  |  |  |  |  |  |
| hnRNPA2B1 | -0.23 | -0.21 |  |  | 0.27 |  |  |  | 0.52 |  |  | 0.26 | 0.43 | -0.48 | 0.28 | -0.37 |  |  |  |
| hnRNPCL1 |  |  |  |  |  |  | 0.54 | 0.27 |  |  |  |  |  |  |  |  |  |  |  |
| hnRNPL |  |  |  |  |  |  |  |  |  |  |  |  |  |  | -0.25 |  |  |  |  |
| hnRNPLL |  |  |  | -0.27 |  |  |  |  |  | -0.45 | -0.37 |  |  |  |  |  |  |  |  |
| hnRNPU |  | -021 |  |  |  | -0.24 |  |  |  |  |  |  |  |  |  |  |  |  |  |
| SRSF1 |  | -0.27 |  |  |  | -0.29 |  |  |  |  |  |  |  | -0.47 |  | -0.41 |  |  | -0.25 |
| SRSF2 | -0.47 | -0.30 | -0.20 |  |  |  | 0.22 |  |  |  |  |  |  |  | -0.87 |  |  |  |  |
| SRSF5 |  |  |  |  |  |  |  |  |  |  |  |  |  |  |  |  |  |  | -0.24 |
| SRSF7 |  | -0.34 | -0.28 |  |  |  | -0.33 | -0.42 | -0.39 | -0.34 | -0.24 | -0.21 |  | -0.29 |  |  |  |  | -0.21 |
| SRSF9 |  | -0.21 |  |  |  |  |  |  |  |  |  |  |  | -0.36 |  | -0.34 |  |  |  |
| SRSF10 | -0.23 | -0.47 |  |  |  | -0.23 |  |  |  |  |  | -0.28 | -0.53 | -0.49 |  |  | -0.33 | -0.56 |  |
| TRA2α |  | -0.22 |  |  |  | -0.28 |  |  |  | -0.21 |  |  |  |  |  |  |  | -0.28 | -0.43 |
| TRA2β | -0.25 | -0.46 |  |  |  |  |  |  |  |  |  |  |  |  |  | -0.21 |  |  | -0.45 |

**Supplementary Table S4.** DeepCLIP analysis of spliceogenic variants with strong impact on splicing (minigene full-length transcript <30% of the overall expression).

Variants with very strong impacts are yellow-shadowed (c.668A>T, c.680C>A and c.680C>T).
