# Supplementary material for: Unproductive alternative splicing of ATM exon 7: mapping of critical regulatory elements and identification of 34 spliceogenic variants
Source: J Mol Med (Berl). 2025 Sep 20;103(11-12):1447–60. doi: 10.1007/s00109-025-02595-0 (PMC12675606; doi:10.1007/s00109-025-02595-0)
Supplement: Supplementary file 8 — Supplementary file8 (DOCX 27 KB) [file 109_2025_2595_MOESM8_ESM.docx]

**Supplementary Table S5.** Tentative ACMG/AMP-based classification of the 19 highly spliceogenic *ATM* exon 7 variants.

| **Variants^1^** | **p.HGVS^1^** | **gnomAD allelle count^2^** | **REVEL^3^** | **Combined minigene-based PVS1_(RNA)/BP7_(RNA) evidence strength^4^** | **minigene-based**  **PVS1_(RNA)/BP7_S(RNA)^4^** | **Final Classification** |
| --- | --- | --- | --- | --- | --- | --- |
| c.667G>C | p.(Glu223Gln) | (-) (PM2) (+1) | 0.189 (BP4) (-1) | P_VS (77%)+B_Supporting (23%) | N/A | VUS (0) |
| c.667G>T | **p.(Glu223Ter)** | (-) (PM2) (+1) | - | **P_VS (100%)** | PVS1_(RNA)+Nonsense (+8) | LP (+9) |
| c.668A>T | p.(Glu223Val) | (-) (PM2) (+1) | 0.343 | **P_VS (87%)**+B_Supporting (13%) | PVS1_(RNA) (+8) | LP (+9) |
| c.669A>T | p.(Glu223Asp) | (-) (PM2) (+1) | 0.109 (BP4) (-1) | P_VS (82%)+B_Supporting (18%) | N/A | VUS (0) |
| c.670A>T | **p.(Lys224Ter)** | (-) (PM2) (+1) | - | **P_VS (100%)** | PVS1_(RNA)+Nonsense (+8) | LP (+9) |
| c.672G>T | p.(Lys224Asn) | (2) (PM2) (+1) | 0.153 (BP4) (-1) | P_VS (74%)+B_Supporting (26%) | N/A | VUS (0) |
| c.677C>T | p.(Ser226Phe) | (-) (PM2) (+1) | 0.036 (BP4) (-1) | P_VS (81%)+B_Supporting (19%) | N/A | VUS (0) |
| c.680C>A | **p.(Ser227Ter)** | (-) (PM2) (+1) | - | **P_VS (97%)**+P_S (3%) | PVS1_(RNA)+Nonsense (+8) | LP (+9) |
| c.680C>T | p.(Ser227Leu) | (1) (PM2) (+1) | 0.063 (BP4) (-1) | **P_VS (96%)**+B_Supporting (4%) | PVS1_(RNA) (+8) | LP (+8) |
| c.871C>A | p.(His291Asn) | (-) (PM2) (+1) | 0.506 | P_VS (74%)+B_Supporting (26%) | N/A | VUS (+1) |
| c.871C>T | p.(His291Tyr) | (-) (PM2) (+1) | 0.49 | P_VS (71%)+B_Supporting (29%) | N/A | VUS (+1) |
| c.877A>T | **p.(Lys293Ter)** | (-) (PM2) (+1) | - | **P_VS (100%)** | PVS1_(RNA)+Nonsense (+8) | LP (+9) |
| c.878A>T | p.(Lys293Ile) | (-) (PM2) (+1) | 0.153 (BP4) (-1) | P_VS (71%)+B_Supporting (29%) | N/A | VUS (0) |
| c.881G>T | p.(Gly294Val) | (1) (PM2) (+1) | 0.469 | P_VS (84%)+B_Supporting (16%) | N/A | VUS (+1) |
| c.882A>G | p.(Gly294=) | (-) (PM2) (+1) | - | P_VS (76%)+B_S (24%) | N/A | VUS (+1) |
| c.882A>T | p.(Gly294=) | (-) (PM2) (+1) | - | P_VS (73%)+B_S (27%) | N/A | VUS (+1) |
| c.892C>T | **p.(Gln298Ter)** | (-) (PM2) (+1) | - | **P_VS (100%)** | PVS1_(RNA)+Nonsense (+8) | LP (+9) |
| c.893A>T | p.(Gln298Leu) | (-) (PM2) (+1) | 0.158 (BP4) (-1) | P_VS (73%)+B_Supporting (27%) | N/A | VUS (0) |
| c.895G>T | **p.(Glu299Ter)** | (-) (PM2) (+1) | - | **P_VS (99%)**+P_S (1%) | PVS1_(RNA)+Nonsense (+8) | LP (+9) |

Variants were classified according to a recently proposed ACMG/AMP point-based classification system, a Bayesian framework that allows for increased flexibility and accuracy in combining different ACMG/AMP criteria and strengths of evidence (PMIDs: 29300386; 32720330). If the variant meets an evidence criterion, the corresponding evidence strengths, according to the point system, are as follows: ±1 (Supporting), ±2 (Moderate), ±4 (Strong), ±8 (Very strong); positive and negative values for pathogenic or benign evidence, respectively. In this framework, point-based variant classification categories are defined as follows: Pathogenic (P) ≥+10; Likely Pathogenic (LP) +6 to +9; Variant of Uncertain Significance (VUS) 0 to +5; Likely Benign (LB) -1 to -6; and Benign (B) ≤-7. To assign ACMG/AMP scores to individual variants, we based our analysis primarily on recently released *ATM* specifications defined by the ClinGen Hereditary Breast, Ovarian and Pancreatic Cancer Variant Curation Expert Panel (<https://clinicalgenome.org/affiliation/50039/>). Evidence not applicable for this study is not shown. Thus, the PM3 code (applied when the variant is detected in Ataxia Telangiectasia patients in trans with a pathogenic variant) was not assigned to any variant.

^1^ HGVS nomenclature using NM_000051.4 as a reference. ^2^ Total allele counts in gnomAD v2.1.1 to assign population frequency code PM2. ^3^ Data used to assign code PP3/BP4. REVEL scores: PP3 ≥0.773 and BP4 ≤0.249. ^4^ Combining evidence strengths to produce an overall PVS1_(RNA)/BP7_(RNA) evidence strength.
